# Frontier molecular orbital effects control the hole-catalyzed racemization of atropisomeric biaryls†
†Electronic supplementary information (ESI) available. See DOI: 10.1039/c8sc05066j


**DOI:** 10.1039/c8sc05066j

**Published:** 2018-12-18

**Authors:** Jacqueline S. J. Tan, Robert S. Paton

**Affiliations:** a Department of Chemistry , Colorado State University , Fort Collins , CO 80523 , USA . http://www.patonlab.com ; Email: robert.paton@colostate.edu; b Chemistry Research Laboratory , University of Oxford , 12 Mansfield Road , Oxford , OX1 3TA , UK

## Abstract

Atropisomeric biaryl systems are privileged architectures used in asymmetric synthesis and pharmaceutical structures. We report that by simply removing a single-electron, the resistance of biaryls towards racemization is reduced dramatically. Even though the steric properties are unaltered, biaryl oxidation changes atropisomerization into a two step mechanism with considerably smaller activation barriers than closed-shell biaryls. The effect is general for a series of biaryls and helicenes studied and results from the dependence of frontier molecular orbital energies on biaryl conformation.

## Abstract 2

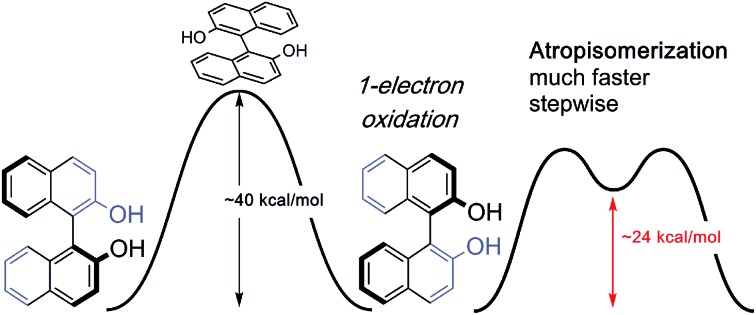
Biaryl atropisomerization is dramatically accelerated by the removal of an electron. The planar transition state is preferentially stabilized from depopulation of the highest occupied molecular orbital.

## Introduction

Axially chiral molecules are of fundamental importance across a range of different fields including catalysis, medicine and materials. Axial chirality can be used as a dynamic means to control reactivity, for example in Click chemistry.^1^ The utility and importance of this property are also exemplified by biaryls such as 1,1′-bi-2-naphthol (BINOL), which represents a privileged architecture in synthetic chemistry.^2^ Kuhn originally introduced the concept of atropisomerism to describe stereoisomers that result from restricted rotation about a single bond that cannot be separated at room temperature.^3^ This was further refined by Ōki, who suggested that atropisomers should interconvert with a half-life of at least 1000 seconds at a given temperature, corresponding to an energy barrier of 22 kcal mol^–1^ at 27 °C.^4^ Such definitions provide a useful “rule of thumb” in considering whether a particular biaryl system exists as stable atropisomers or to the contrary, is amenable to racemization. In a pharmaceutical setting, guidelines have been developed for atropisomeric molecules, where half-lives ranging from days to weeks are preferred to avoid racemization *in vivo*.^5^ Rotational barriers in excess of 29.4 kcal mol^–1^ are therefore recommended.

Atropisomerization of biaryls is an intramolecular process controlled by substituent sterics, and to a lesser extent, by electronic effects.^6^ However, racemization can also be promoted under acidic conditions,^7^ which proceeds *via* dearomatized protonated intermediates, and under basic conditions,^8^ due to deprotonation and dianion formation.

**Scheme 1:**
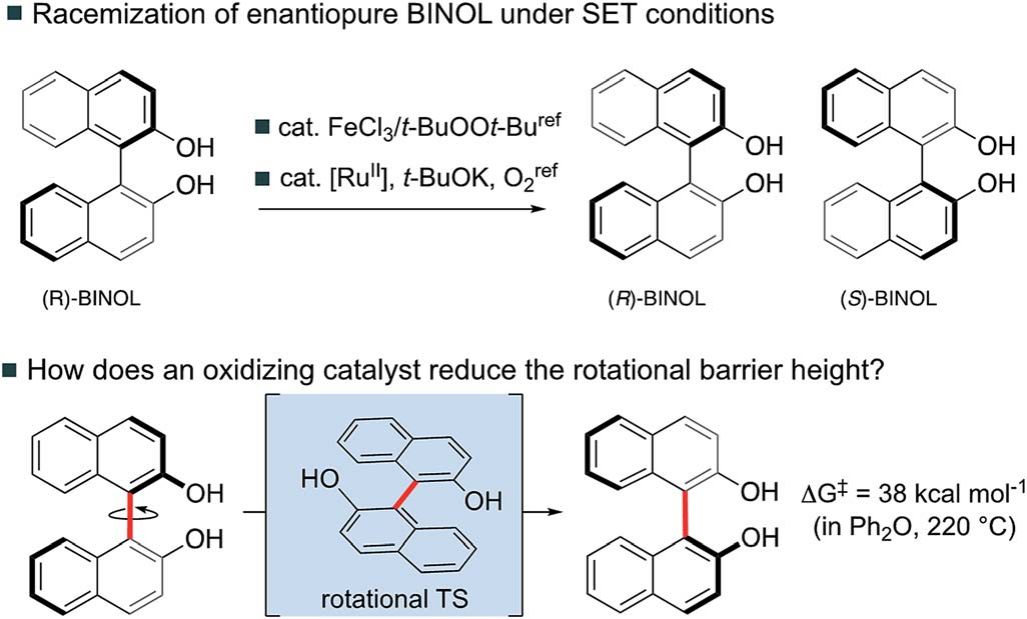
BINOL racemization is observed under single-electron-transfer (SET) conditions at temperatures for which atropisomers do not interconvert.

Photolysis experiments also result in facile racemization by accessing the excited triplet state.^9^ More recently, however, Pappo has reported that optically pure BINOL derivatives undergo racemization at room temperature under single-electron-transfer (SET) conditions (Scheme 1).^10^ Similar SET conditions have been recently reported by Akai to promote biaryl racemization at 35–50 °C, which underpins the development of an enzymatic dynamic kinetic resolution of biaryls in the same laboratory.^11^ Chen has also found that oxidation with a hypervalent iodine reagent promotes BINOL racemization.^12^ The precise mechanistic origins to explain why biaryl oxidation should render more facile axial rotation, leading to easier racemization, remain unclear. In recent theoretical studies, calculations have provided usefully accurate predictions of racemization rate constants.^13^ The racemization mechanism of helicenes have also been reviewed recently using DFT methods.^14^ Given the typically high resistance of BINOL towards thermal racemization (the barrier has been determined experimentally as 37–38 kcal mol^–1^ such that temperatures above 200 °C are required),^15^ SET clearly induces a substantial barrier-lowering effect in the rotational transition state (Scheme 1). We therefore set out to uncover the origins of this catalytic atropisomerization in a computational study.

## Results and discussion

**Fig. 1:**
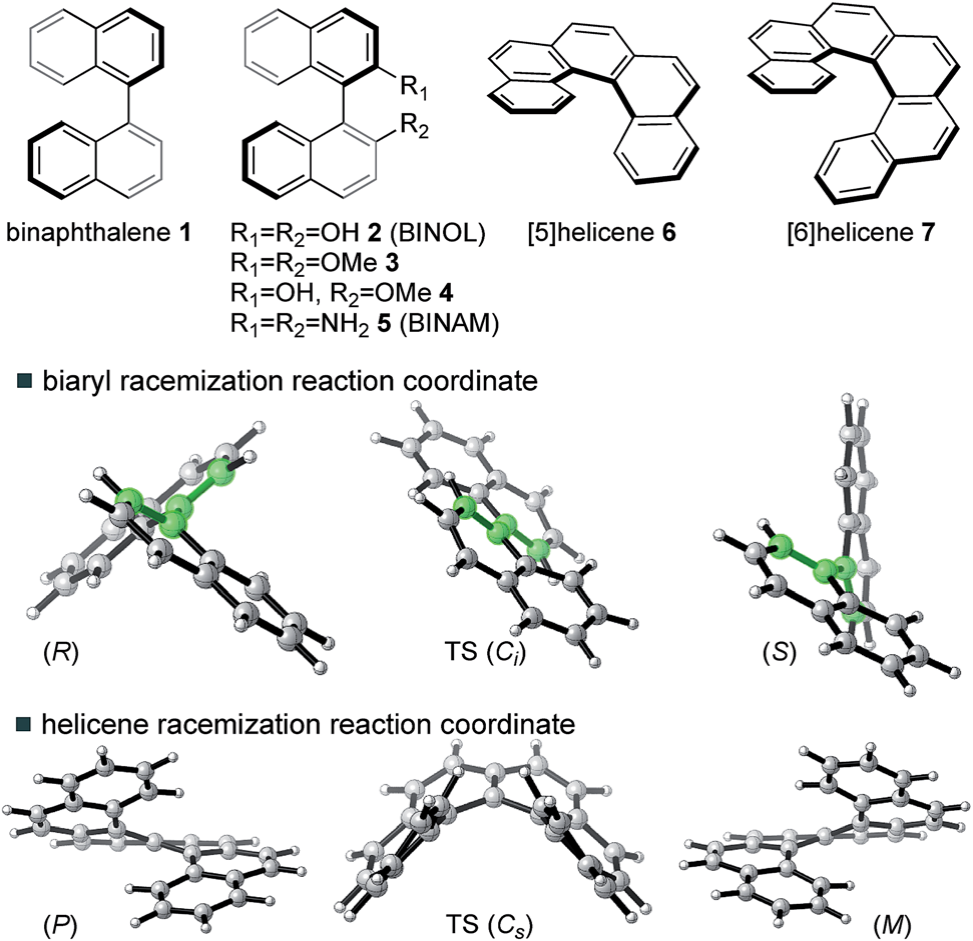
Atropisomeric compounds studied computationally.

We have computed the effects of the removal of a single electron upon the rotational barriers, and hence racemization/atropisomerization of, various biaryl systems. Although catalyst coordination may play a role in this process, we focus our attention on the intrinsic effect of hole-catalysis^16^ in forming an oxidized biaryl molecule: as we shall discuss, this effect turns out to be substantial and, we suggest, a crucial aspect of this chemistry. We compare racemization barriers for these radical-cationic systems^17^ against those obtained for the parent, neutral system. In addition to a series of binaphthyl and BINOL derivatives we have also included helicenes in our study since these exhibit similar thermal barriers towards atropisomerization, albeit not *via* the rotation of a single C–C bond (Fig. 1).

Our investigations use density functional theory (DFT), for which we found extremely good reproduction of experimentally determined rotational barriers for several neutral systems. B3LYP-D3(BJ)/def2-TZVP//B3LYP/6-31G(d) and M06-2X/def2-TZVP//M06-2X/6-31G(d) calculations gave barrier heights within 2 kcal mol^–1^ of experiment (see ESI Table S2 and S3†).^18^ The effects of solvation in dichloromethane were included implicitly by SMD calculations and had a very small effect (typically <1 kcal mol^–1^) on the rotational barriers. Full details of all calculations are given in the ESI;† in the main text we refer to B3LYP-D3 results, although there is little qualitative or quantitative difference from M06-2X. Understanding the chemistry of radical cations has benefited considerably from quantum mechanical calculations.^19^ However, DFT descriptions of radical cation dissociation are known to fail due to the effects of delocalization error (also known as self-interaction or charge-transfer error).^20^ Consistent results were obtained in our study using functionals with different amounts of exact Hartree–Fock exchange (which were also replicated for **1˙^+^** with a range-separated hybrid, LC-ωPBE), so these density-driven errors do not appear to influence the nature of our conclusions.^21^ The calculations were additionally performed in solvent (dichloromethane), which did not differ from the gas phase results.

Fig. 1 illustrates the characteristic transition structures for the interconversion of chiral biaryl and helicene forms for closed-shell systems.^15^ Axial rotation from the ground state (GS) of the biaryls can proceed in either sense, however, the *anti*-TS shown (in which the 8-positions are as far apart as possible) is preferred for steric reasons.^15^ For helicenes, racemization between (*P*) and (*M*) configurations proceeds *via* a puckered TS. A structural comparison of ground state (GS) and transition structure (TS) for neutral and one-electron oxidized biaryl systems shows some clear differences (Table 1). After oxidation, the biaryl C–C bond is shortened in the GS 0.03–0.04 Å and by up to 0.05 Å in the TS. Initially this is surprising, since unfavourable H–H steric interactions in the racemization TS should be even more severe for the radical cation, leading to a qualitative prediction that racemization is slowed upon oxidation. However, computed activation barriers show that this is decidedly not the case, with a very large barrier-lowering effect of 8–14 kcal mol^–1^ due to oxidation (ESI Table S2†).

**Table 1:** Biaryl bond lengths and dihedral angles in the ground state and in the rotational transition state structure (B3LYP/6-31G(d))

Species			C–C bond length/Å	Dihedral angle/°
**1**	Neutral	GS	1.50	105
		TS	1.50	180
	Radical cation	GS	1.46	125
		TS	1.45	180
**2**	Neutral	GS	1.50	95
		TS	1.49	180
	Radical cation	GS	1.47	115
		TS	1.47	143
**3**	Neutral	GS	1.50	88
		TS	1.48	180
	Radical cation	GS	1.47	65
		TS	1.43	175
**4**	Neutral	GS	1.50	86
		TS	1.49	173
	Radical cation	GS	1.47	145
		TS	1.46	180
**5**	Neutral	GS	1.50	88
		TS	1.48	179
	Radical cation	GS	1.47	111
		TS	1.46	145

**Fig. 2:**
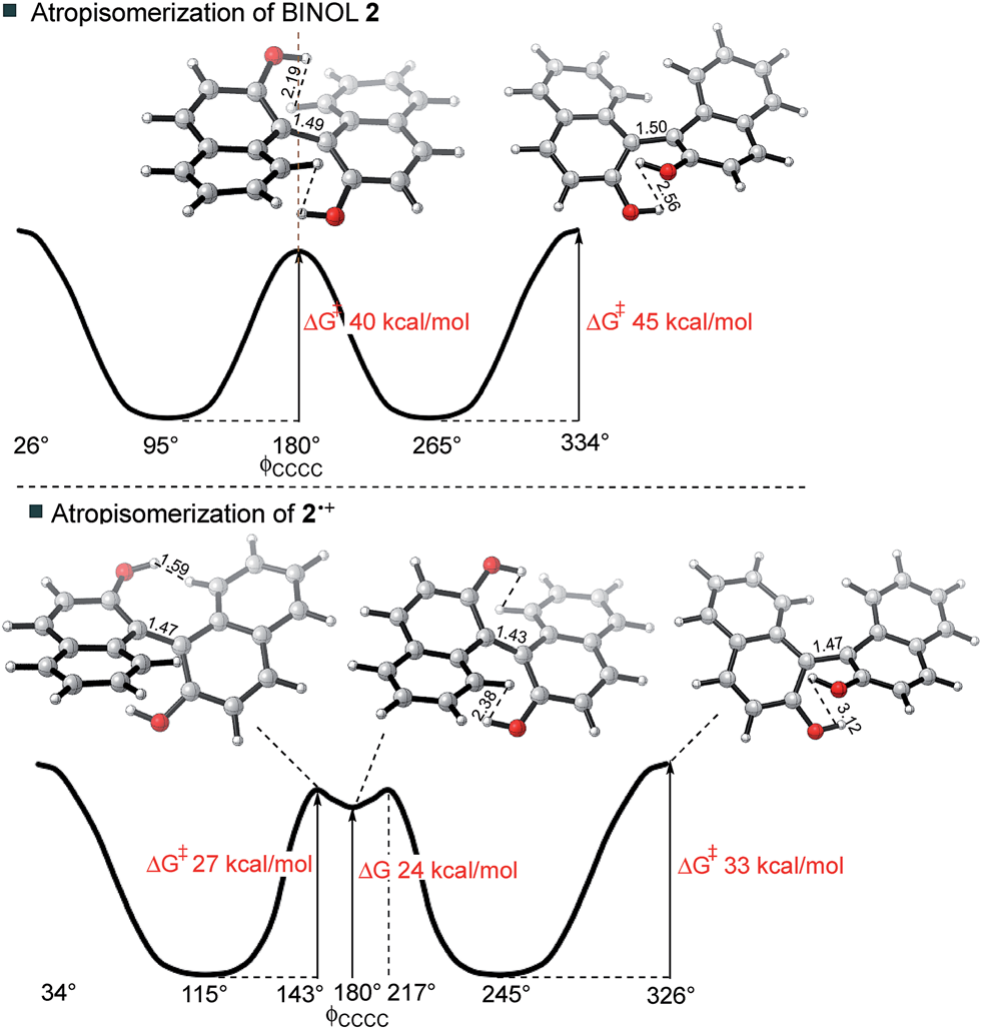
The lowest energy thermal racemization of BINOL **2** proceeds *via* a single achiral transition structure (TS); the racemization of **2˙^+^** proceeds *via* two TSs and an achiral intermediate with a much lower overall activation barrier.

For neutral compounds, naphthyl rings are close to perpendicular in the GS (dihedral angles close to 90°), and parallel in the TS (dihedral angles of 180°). For the radical cations, this distinction is less clear: in fact, the radical TSs are twisted from planarity (dihedral angles < 180°). Rotation about the central biaryl axis can occur in either sense, which gives rise to two distinct enantiomerization pathways (*anti* and *syn*). As shown in Fig. 2, the *anti*-pathway is generally favored by more than ∼5 kcal mol^–1^, and we focus our attention on this preferred sense of rotation henceforth. For the closed-shell biaryls, the rotational *anti*-TS is centrosymmetric and hence achiral. To our surprise, we found the reaction coordinate for radical cation biaryls actually involves two steps, *via* two degenerate rotational TSs which are both chiral (Fig. 2). An intervening shallow intermediate (lying *ca.* 2 kcal mol^–1^ below these structures) is the centrosymmetric species which breaks chirality.

Racemization of a closed-shell biaryl (*e.g.* BINOL **2**) proceeds *via* a high barrier in a single-step. The reaction coordinate for the **2˙^+^** is more complex: the rotational TS (of which there are two mirror image forms) is unsymmetrical and more twisted (the central dihedral being 143° rather than 180°). The symmetrical (shallow) intermediate resembles the closed-shell TS, although with a shorter central bond and closer non-bonding interactions. It is, however, much more stable than the closed-shell TS by around 15 kcal mol^–1^! As we will show, the shorter steric contacts are more than compensated for by the increased bonding interaction of the connecting C–C biaryl bond.

The energetic consequences of single-electron oxidation (Table 2), are clear: a reduction in racemization barrier height relative to the neutral compounds is seen in every example studied. These effects are substantial – biaryl rotational barriers are reduced by 8–14 kcal mol^–1^ and helicene barrier by 5–6 kcal mol^–1^, corresponding to an enhancement in racemization rate by many orders of magnitude. We predict that the racemization temperatures (required for a *t*_1/2_ of ∼10^3^ s) for BINOL derivatives and BINAM (**2–5**) will drop by 200 °C! Although the oxidized compounds (**2–5**) still have barriers in excess of that suggested by Ōki to be considered as atropisomeric at room temperature,^4^ racemization is now accessible at temperatures attainable in many organic solvents (<100 °C), unlike their parent compounds. The measured oxidation potentials for several of the compounds studied suggest that one-electron oxidation is feasible with a range of oxidants.

**Table 2:** B3LYP-D3/def2TZVP//B3LYP/6-31G* computed and experimental activation parameters for the racemization of compounds **1–7**

**Molecule**	**1**	**2**	**3**	**4**	**5**	**6**	**7**
Experimental barrier (kcal mol^–1^)	24.1^*a*^	37.8^*a*^	—	—	40.9^*b*^	24.1^*c*^	36.2^*c*^
Calculated barrier (kcal mol^–1^)	24.6	39.9	39.6	39.9	42.4	24.4	37.3
Racemization temp. (K)	336	538	534	538	571	334	504
Half-life *t*_1/2_ at rt (hours)	69	10^13^	10^13^	10^13^	10^15^	50	10^12^
*E*° potential/V	0.65^*d*^	1.18^*d*^	0.68^*e*^	—	—	1.14^*c*^	1.08^*c*^
**Radical cation**	**1˙^+^**	**2˙^+^**	**3˙^+^**	**4˙^+^**	**5˙^+^**	**6˙^+^**	**7˙^+^**
Calculated barrier (kcal mol^–1^)	16.9	26.6	25.6	26.1	28.0	19.7	31.7
Racemization temp. (K)	232	363	349	356	381	271	430
Half-life *t*_1/2_ at rt (hours)	10^–4^	2310	379	994	2 × 10^4^	0.015	10^7^

**ΔΔ*G*** ^**‡**^ **(kcal mol** ^**–1**^ **)**	**7.8**	**13.3**	**14.1**	**13.8**	**14.4**	**4.7**	**5.6**
Relative racemization rate, *k*_rel_	7 × 10^5^	9 × 10^9^	3 × 10^10^	2 × 10^10^	5 × 10^10^	2 × 10^3^	2 × 10^3^

^*a*^ Ref. 15.

^*b*^ Ref. 22.

^*c*^ Ref. 2b; *E*° *vs.* SHE.

^*d*^ Ref. 23, *E*° *vs.* Ag/AgCl in CH_2_Cl_2_/CHCl_3_–BFEE.

^*e*^ Ref. 5, *E*° *vs.* Ag/AgCl in CH_2_Cl_2_/CHCl_3_–BFEE.

**Fig. 3:**
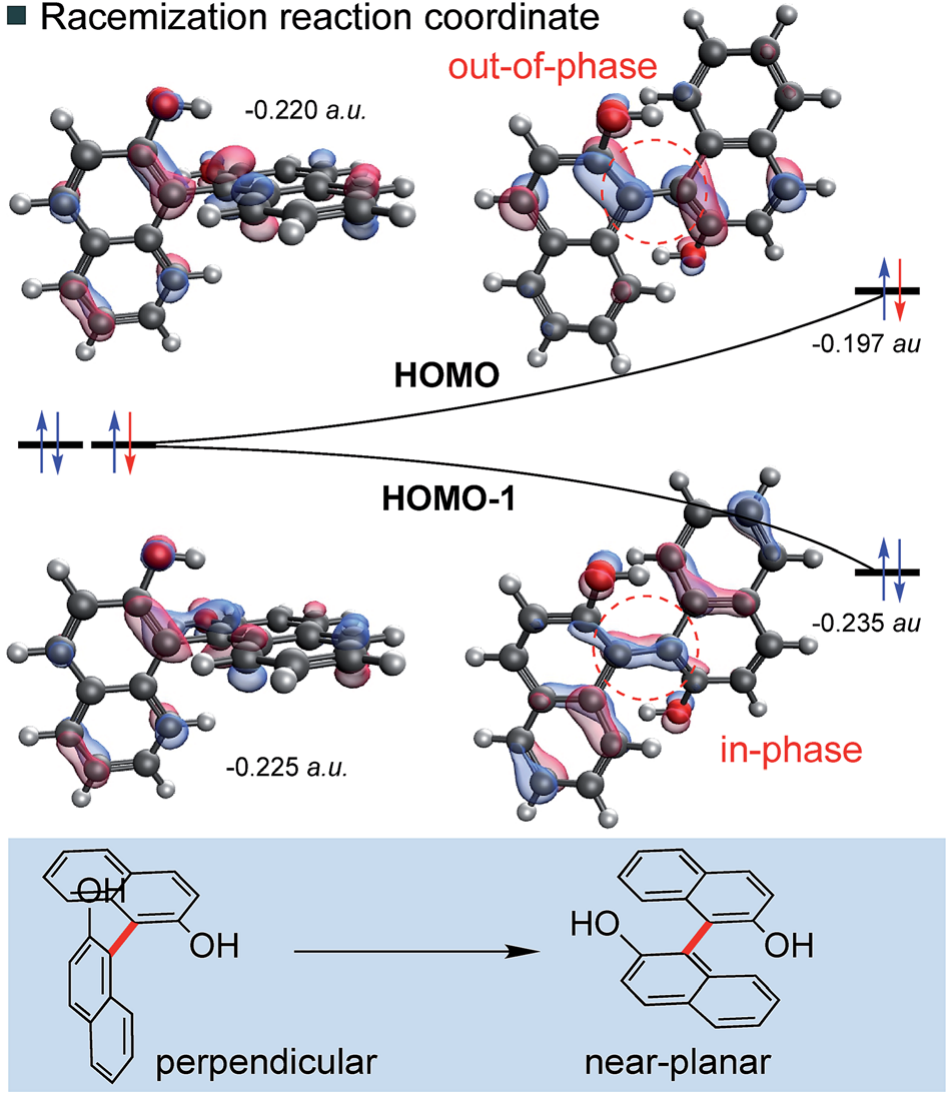
Involvement of frontier molecular orbitals (FMOs) in biaryl racemization. The orbital correlation diagram shows the B3LYP/def2TZVP energies of the two highest occupied MOs.

**Fig. 4:**
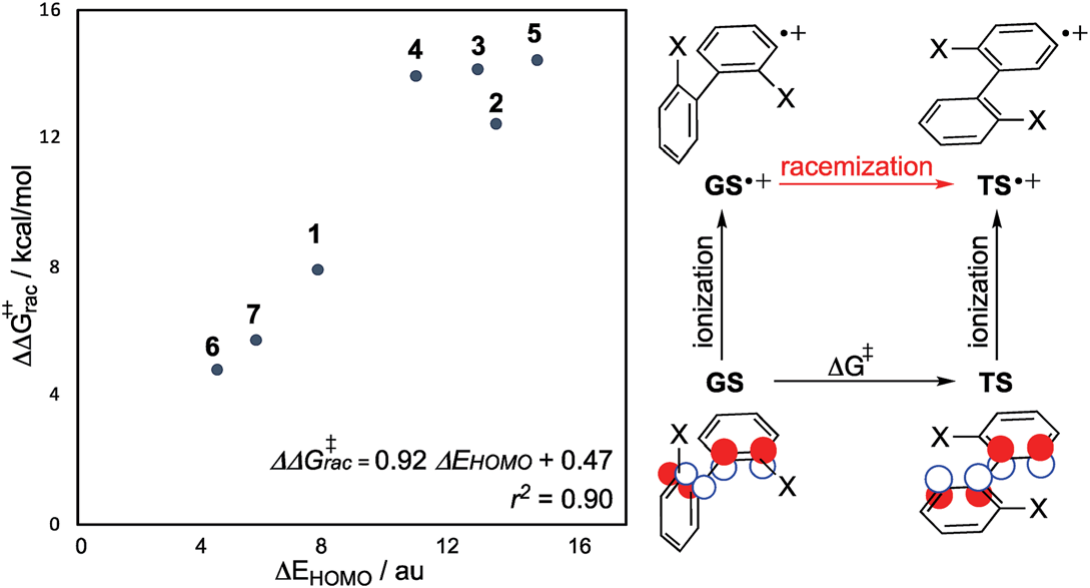
The change in HOMO energy level between twisted and planar biaryl conformations is a good predictor of the racemization barrier-lowering effect from SET (note the different energy units for *x* and *y* axes).

The steric properties of each biaryl are unaltered by oxidation, and so logically, the increase in the rate of enantiomerization is caused by changes in electronic structure. Indeed, we have discovered the origins are rooted in frontier molecular orbital (FMO) theory, and furthermore, can be explained with an orbital correlation diagram showing the two highest occupied molecular orbitals (HOMOs) (Fig. 3). A focus on frontier orbitals may seem counterintuitive for a reaction in which bond-formation is totally absent! However, as we shall show, our model rationalizes all structural and energetic observations and even provides good quantitative predictions of the reduction in barrier height (Fig. 4).

Biaryl HOMOs are formed from a combination of the two HOMOs of the individual naphthalene π-systems. In a ground state conformation these two naphthalenes are (close to) perpendicular, such that there is no (or very little) interaction between the orthogonal π-systems: accordingly, there are two degenerate HOMOs, shown in Fig. 3. An equivalent representation would show one HOMO on each ring system. Racemization results from rotation about the biaryl bond, so that the naphthalene π-systems are now able to interact as the conformation approaches planarity. The HOMO and HOMO-1 energies diverge as a result of the in-phase (bonding) and out-of-phase (anti-bonding) overlap across the biaryl C–C bond. Very simply, the removal of an electron from biaryls stabilizes the planar transition state by lessening the impact of a raise in HOMO energy. This energetic cost is halved, since the orbital is singly- rather than doubly-occupied.

This logic explains several observations. Removing an electron from the HOMO stabilizes the planar TS to the extent that it becomes a minimum on the potential energy surface (Fig. 2). The shortening of the central biaryl bond of radical cations, as seen particularly in the rotational TS and achiral minimum, is a result of greater bonding character between the two carbon atoms (Fig. 3). The HOMO has anti-bonding overlap between these two atoms: removal of an electron therefore increases the local bond order in this region. This orbital could also be depopulated *via* photochemical means, bringing a similar change in the bond order as well.^24^ Two electrons remain in the HOMO–1, which has a bonding interaction here. Furthermore, the preference of radical cations to adopt more planar structures in the GS than the corresponding neutral systems is again a consequence of the stabilization gained from the net-bonding interaction between the two naphthalene π-systems when the HOMO is no longer doubly-occupied.

We reasoned that a quantitative relationship should exist between the conformational dependence of the biaryl HOMO energy and the reduction in barrier height from one-electron oxidation. The energy change between orthogonal and planar conformations for closed-shell and radical cation systems can be formally related using a thermodynamic cycle (Fig. 4). Acknowledging Koopmans' theorem, which connect the ionization energies to the negative of the HOMO energies, the change in activation barrier ΔΔ*G*^‡^ following oxidation is equal to the change in (closed-shell) HOMO energy between the GS and TS. This predictive model, which contains information only from the closed-shell species, describes the barrier-lowering effect well (Fig. 4, *r*^2^ = 0.90) from single-electron oxidation. This lends further support to our FMO considerations. It is also noticeable that the helicenes have a smaller reduction in energy barrier, and a smaller ΔHOMO, which is reasonable as the TS of helicenes are puckered and not coplanar like the biaryls.

## Conclusions

We have described the first theoretical studies on how one-electron oxidation affects biaryl atropisomerism, and consequently, the rate of racemization. Following oxidation, the resistance towards biaryl rotation is dramatically reduced. The planar conformation of oxidized biaryls undergo preferential stabilization, such that the rotational transition state of closed-shell biaryls becomes a stable minimum and the enantiomerization mechanism changes from one, to two-steps. We predict that singly-oxidized biaryls can undergo racemization at temperatures far below those required for the parent compound: such atropisomerization may also be assisted by metal coordination. Single electron transfer from biaryls preferentially stabilizes the planar form over the non-planar ground state since the HOMO energy is higher in this species, which creates a greater bonding interaction across the connecting biaryl bond. We hypothesize that this effect may find use in the development of new methods for the asymmetric synthesis of biaryls,^25^ particularly if it can be harnessed in the context of dynamic kinetic resolution.^11^ Additionally, oxidatively-promoted biaryl and helicene racemization may have implications for the application of such compounds in pharmaceutical applications.

## Conflicts of interest

There are no conflicts to declare.

## Supplementary Material

Supplementary information
