# Supplementary material for: Frontier molecular orbital effects control the hole-catalyzed racemization of atropisomeric biaryls
Source: Chem Sci. 2018 Dec 18;10(8):2285–9. doi: 10.1039/c8sc05066j (PMC6399675; doi:10.1039/c8sc05066j)
Supplement: Supplementary file 1 [file SC-010-C8SC05066J-s001.pdf]

Electronic Supplementary Material (ESI) for Chemical Science.
This journal is © The Royal Society of Chemistry 2018

Supplementary Materials for

Frontier Molecular Orbital Occupancy Controls Hole-Catalyzed Racemization of Atropisomeric Biaryls

Jacqueline S. J. Tan[†], Robert S. Paton[*‡†]

[‡] Department of Chemistry, Colorado State University, Fort Collins, CO 80523, USA

[†] Chemistry Research Laboratory, 12 Mansfield Road, Oxford OX1 3TA, UK

robert.paton@colostate.edu

Table of Contents

1. **Quantum Chemical Calculations** S2
2. **Comparison of B3LYP and M06-2X Results** S2
3. **Absolute Contributions to Gibbs Energies** S4
4. **Cartesian Coordinates** S8
5. **References** S27

1. Quantum Chemical Calculations

Density functional theory (DFT) calculations were performed with Gaussian 09 rev. D.01.[1] All singlet and doublet species were described with unrestricted Kohn-Sham theory; converged densities were checked for their stability (*stable=opt*). Geometry optimisations used the B3LYP[2] and M06-2X[3] functionals with 6-31G(d) basis set for all atoms. Single point energies corrections were done using B3LYP-D3(BJ)[4] with the def2-TZVP basis set,[5] and M06-2X with an ultrafine grid. Solvation in dichloromethane is employed via single-point calculations with Solvent Model Based on Density Model (SMD).[6]

Minima and transition structures on the potential energy surface (PES) were confirmed as such by harmonic frequency analysis at the same level of theory. All transition structures were verified with intrinsic reaction coordinate (IRC) calculations.[7] All spin density plots have been generated at 0.15 isovalue using *IQmol*.[8]

The reaction considered is unimolecular and so a standard state correction (from 1 atm to 1 mol/l) has no effect. Gibbs energies were evaluated at 298.15 K, using a quasi-RRHO treatment of vibrational entropies introduced by Grimme.[9] Vibrational entropies of frequencies below 100 cm^{-1} were obtained according to a free rotor description, using a smooth damping function to interpolate between the two limiting descriptions. This was implemented using Goodvibes.[10]

2. Comparison of B3LYP and M06-2X Results

Table S1. Bond lengths and dihedral angles of 5 biaryl systems at B3LYP-D3/def2TZVP//B3LYP/6-31G* and M062X/def2TZVP//M062X/6-31G*.

Molecule	Reaction	Energy Level	Bond length ℓ/Å	Dihedral angle/°
	**Neutral**	GS	1.497/1.491	-105/-112
**1**		TS	1.500/1.499	180/180
	**Radical cation**	GS	1.464/1.452	-125/-127
		TS	1.454/1.446	-180/-168
	**Neutral**	GS	1.498/1.490	-95/-112
**2**		TS	1.486/1.487	180/180
	**Radical cation**	GS	1.467/1.452	-115/-119
		TS	1.466/1.461	-143/-137
	**Neutral**	GS	1.496/1.489	-88/-70
**3**		TS	1.482/1.488	180/174
	**Radical cation**	GS	1.471/1.454	-65/-59
		TS	1.429/1.431	-175/-167
	**Neutral**	GS	1.498/1.491	-86/-69
**4**		TS	1.485/1.489	-173/-170
	**Radical cation**	GS	1.471/1.471	145/137
		TS	1.461/1.459	-180/-168
	**Neutral**	GS	1.499/1.492	-88/-71
**5**		TS	1.477/1.480	-179/-175
	**Radical cation**	GS	1.472/1.460	-111/-64
		TS	1.460/1.458	-145/-138

Table S2. ΔG values calculated at the B3LYP-D3/def2TZVP//B3LYP/6-31G* level of theory, compared with experimental values and E°(solvent effects calculated from SMD represented in parentheses)

**Molecule**	**1**	**2**	**3**	**4**	**5**	**6**	**7**
Experimental barrier (kcal/mol)	24.1[a]	37.8[a]	-	-	40.9[b]	24.1[c]	36.2[c]
Calculated barrier (kcal/mol)	24.6	39.9	39.6	39.9	42.4	24.4	37.3
Racemization Temp. (K)	336	538	534	538	571	334	504
Half-life $t_{1/2}$ at rt (hours)	69	10^{13}	10^{13}	10^{13}	10^{15}	50	10^{12}
E° Potential/V	0.65[d]	1.18[d]	0.68[e]	-	-	1.14[c]	1.08[c]
**Radical cation:**	$\mathbf{1}^{\cdot+}$	$\mathbf{2}^{\cdot+}$	$\mathbf{3}^{\cdot+}$	$\mathbf{4}^{\cdot+}$	$\mathbf{5}^{\cdot+}$	$\mathbf{6}^{\cdot+}$	$\mathbf{7}^{\cdot+}$
calculated barrier (kcal/mol)	16.9	26.6	25.6	26.1	28.0	19.7	31.7
Racemization Temp. (K)	232	363	349	356	381	271	430
Half-life $t_{1/2}$ at rt (hours)	10^{-4}	2310	379	994	$2x10^{4}$	0.015	10^{7}
ΔΔG‡ **(**kcal/mol)	7.8	13.3	14.1	13.8	14.4	4.7	5.6
Relative racemization rate, k_{rel}	7×10^{5}	9×10^{9}	3×10^{10}	2×10^{10}	5×10^{10}	$2 x10^{3}$	2×10^{3}

[a] Ref. 13 [b] Ref. 19 [c] Ref. 1b; E° vs. SHE [d] Ref. 20, E° vs. Ag/AgCl in $CH_2Cl_2/CHCl_3$–BFEE [e] Ref. 4, E° vs. Ag/AgCl in $CH_2Cl_2/CHCl_3$–BFEE

Table S3. ΔG values calculated at the M062X/def2TZVP//M062X/6-31G* level of theory, compared with experimental values and E°(solvent effects calculated from SMD represented in parentheses)

**Molecule**	**1**	**2**	**3**	**4**	**5**	**6**	**7**
Experimental/kcal/mol	24.1[a]	37.8[a]	-	-	40.9[b]	24.1[c]	36.2[c]
Neutral ΔG/kcal/mol	25.4(24.7)	40.3(40.6)	39.5(38.9)	40.0(40.0)	43.1(43.0)	25.8(24.2)	38.4(37.2)
Racemization Temperature (for $t_{1/2}$=16.7min)/K	**347(337)**	**543(547)**	**533(526)**	**539**	**580**	**352(332)**	**518(502)**
Half-life $t_{1/2}$ (at 293K)/hours	*299(93)*	*10^{13}*	*$10^{13}(10^{12})$*	*10^{13}*	*10^{15}*	*509(36)*	*$10^{12}(10^{11})$*
E° Potential/V	0.65[d]	1.18[d]	0.68[e]	-	-	1.14[c]	1.08[c]
**Radical cation** ΔG/kcal/mol	16.6(16.0)	26.3(26.0)	24.4(23.7)	24.6(25.2)	27.8(26.4)	21.2(20.2)	32.6(31.6)
Racemization Temperature (for $t_{1/2}$=16.7min)/K	**229(222)**	**359(355)**	**334(325)**	**336(347)**	**378(360)**	**291(278)**	**442(429)**
Half-life $t_{1/2}$ (at 293K)/hours	*10^{-5}*	*1400(826)*	*54.2(13.8)*	*70.3(21.2)*	*$10^{4}(10^{3})$*	*0.21(0.04)*	*$10^{8}(10^{7})$*
ΔΔG‡/kcal/mol	8.8(8.7)	14.0(14.6)	15.1(15.2)	15.4(14.8)	15.3(16.6)	4.5(4.0)	5.8(5.6)
k_{rel}	*10^{6}*	*10^{10}*	*10^{11}*	*10^{11}*	*$10^{11}(10^{12})$*	*10^{3}*	*10^{4}*

[a] Ref. 10 [b] Ref. 2 [c] Ref. 9, E° vs. SHE [d] Ref. 7, E° vs. Ag/AgCl in $CH_2Cl_2/CHCl_3$–BFEE [e] Ref. 8, E° vs. Ag/AgCl in $CH_2Cl_2/CHCl_3$–BFEE

Table S4. HOMO and HOMO-1 energy values at B3LYP-D3/def2TZVP//B3LYP/6-31G*, compared with ΔΔG from Table S2.

**Molecule**	**1**	**2**	**3**	**4**	**5**	**6**	**7**
**HOMO/au(GS)**	-0.216	-0.220	-0.205	-0.209	-0.202	-0.213	-0.211
**HOMO/au(TS)**	-0.203	-0.197	-0.183	-0.190	-0.177	-0.206	-0.201
**ΔHOMO/kcal/mol**	*8.321*	*14.452*	*13.843*	*11.722*	*15.876*	*4.832*	*6.181*
**ΔΔG/kcal/mol**	*7.803*	*12.332*	*14.065*	*13.818*	*14.357*	*4.738*	*5.639*
**HOMO-1/au(GS)**	-0.230	-0.225	-0.206	-0.219	-0.203	-0.218	-0.213
**HOMO-1/au(TS)**	-0.240	-0.235	-0.217	-0.225	-0.211	-0.224	-0.212
**ΔHOMO-1/kcal/mol**	-6.118	-6.557	-7.241	-3.916	-4.750	-3.759	1.117

3. Absolute Contributions to Gibbs Energies

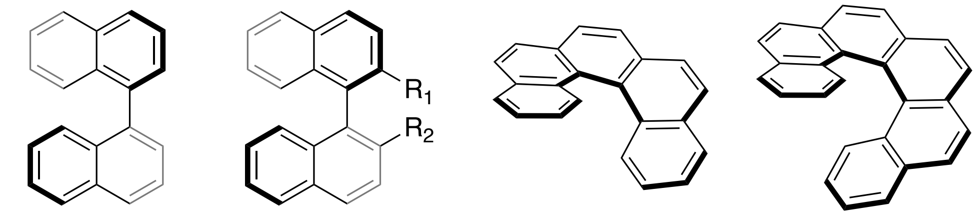

binaphthalene **1** R$_1$=R$_2$=OH **2** (BINOL) [5]helicene **6** [6]helicene **7**

R$_1$=R$_2$=OMe **3**

R$_1$=OH, R$_2$=OMe **4**

R$_1$=R$_2$=NH$_2$ **5** (BINAM)

		E (au)	ZPE (au)	H (au)	T.qh-S (au)	qh-G(T) (au)	imag. ν (cm^{-1})	single point energy (au)
**Molecule 1**				**B3LYP/6-31G(d)**				**B3LYP-D3(BJ)/def2-TZVP**
Neutral	GS	-770.58763	0.275673	-770.29683	0.055312	-770.35215		-770.943581
	TS	-770.55071	0.276376	-770.26031	0.05267	-770.31298	-39.78	-770.90665
Radical cation	GS	-770.33696	0.276222	-770.04567	0.055464	-770.10113		-770.682671
	TS	-770.31095	0.27651	-770.02033	0.053488	-770.07382	-10.17	-770.657227
**Molecule 1**				**M06-2X/6-31G(d)**				**M06-2X/def2-TZVP**
Neutral	GS	-770.25958	0.278497	-769.96607	0.054993	-770.02107		-770.533922
	TS	-770.22151	0.279225	-769.92835	0.052517	-769.98087	-37.71	-770.495502
Radical cation	GS	-769.99217	0.278538	-769.69865	0.055287	-769.75394		-770.258795
	TS	-769.96677	0.278836	-769.67387	0.053451	-769.72732	-22.98	-770.233506

**Molecule 2**				**B3LYP/6-31G(d)**				**B3LYP-D3(BJ)/def2-TZVP**
Neutral	GS	-921.02799	0.284105	-920.72649	0.060005	-920.78649		-921.469161
	TS (anti)	-920.96767	0.28388	-920.66769	0.056405	-920.72409	-3.53	-921.407547
	TS (syn)	-920.96121	0.28420	-920.66096	0.055739	-920.71669	-18.74	-921.400852
Radical cation	GS	-920.77558	0.28443	-920.47385	0.059732	-920.53358		-921.206009
	INT (180°)	-920.73633	0.284339	-920.43481	0.060507	-920.49531		-921.166491
	TS (anti)	-920.73427	0.284262	-920.43358	0.057869	-920.49145	-33.12	-921.164378
	TS (syn)	-920.72449	0.284251	-920.42383	0.057239	-920.48106	-42.46	-920.911118
**Molecule 2**				**M06-2X/6-31G(d)**				**M06-2X/def2-TZVP**
Neutral	GS	-920.65748	0.287483	-920.35282	0.058874	-920.41169		-921.009693
	TS (anti)	-920.59681	0.287693	-920.29325	0.056004	-920.34926	-5.32	-920.94716
	TS (syn)	-920.59078	0.287903	-920.28702	0.055428	-920.34245	-25.64	-920.941045
Radical cation	GS	-920.39009	0.2874	-920.08559	0.059314	-920.1449		-920.733478
	INT (180°)	-920.35370	0.287743	-920.04903	0.059169	-920.10820		-920.696678
	TS (anti)	-920.34857	0.287004	-920.04507	0.058086	-920.10316	-53.22	-920.691721
	TS (syn)	-920.3386	0.287342	-920.03495	0.057087	-920.09203	-54.02	-920.681921

Molecule 3	B3LYP/6-31G(d)							B3LYP-D3(BJ)/def2-TZVP
Neutral	GS	-999.62675	0.340808	-999.26541	0.066127	-999.33154		-1000.094059
	TS	-999.56307	0.340176	-999.20364	0.062783	-999.26643	-11.06	-1000.032309
Radical cation	GS	-999.39095	0.341348	-999.02906	0.066359	-999.09542		-999.847882
	TS	-999.34911	0.341049	-998.98862	0.063925	-999.05255	-7.9	-999.808152
**Molecule 3**	**M06-2X/6-31G(d)**							**M06-2X/def2-TZVP**
Neutral	GS	-999.21144	0.344973	-998.84623	0.065205	-998.91144		-999.581851
	TS	-999.15114	0.344522	-998.78771	0.06211	-998.84982	-16.06	-999.520162
Radical cation	GS	-998.96099	0.344895	-998.59581	0.065776	-998.66158		-999.322468
	TS	-998.92366	0.345035	-998.55945	0.06339	-998.62284	-18.92	-999.284919

Molecule 4	B3LYP/6-31G(d)							B3LYP-D3(BJ)/def2-TZVP
Neutral	GS	-960.32693	0.312371	-959.99554	0.063032	-960.05857		-960.781292
	TS	-960.26496	0.312056	-959.9352	0.059664	-959.99486	-22.85	-960.719353
Radical cation	GS	-960.08369	0.312872	-959.75188	0.063108	-959.81499		-960.527362
	TS	-960.04214	0.312657	-959.71148	0.061055	-959.77254	-23.65	-960.486599
**Molecule 4**	**M06-2X/6-31G(d)**							**M06-2X/def2-TZVP**
Neutral	GS	-959.93397	0.316085	-959.59909	0.062239	-959.66133		-960.295308
	TS	-959.87327	0.315869	-959.5399	0.059323	-959.59922	-27.22	-960.233
Radical cation	GS	-959.6759	0.31605	-959.3411	0.062699	-959.4038		-960.026283
	TS	-959.63527	0.31578	-959.30146	0.061158	-959.36261	-47.04	-959.987638

Molecule 5	B3LYP/6-31G(d)							B3LYP-D3(BJ)/def2-TZVP
Neutral	GS	-881.29642	0.308834	-880.96956	0.060434	-881.02999		-881.720857
	TS	-881.2301	0.308394	-880.90486	0.057528	-880.96239	-2.67	-881.654594
Radical cation	GS	-881.06716	0.309007	-880.73988	0.061378	-880.80126		-881.480627
	TS	-881.02434	0.309626	-880.69774	0.058664	-880.7564	-32.54	-881.437994
**Molecule 5**	**M06-2X/6-31G(d)**							**M06-2X/def2-TZVP**
Neutral	GS	-880.93108	0.312137	-880.60112	0.059852	-880.66098		-881.261563
	TS	-880.86506	0.312086	-880.53639	0.057095	-880.59349	-9.73	-881.194296
Radical cation	GS	-880.68865	0.312244	-880.35847	0.060666	-880.41914		-881.010044
	TS	-880.64662	0.312871	-880.3169	0.058501	-880.3754	-45.25	-880.967469

Molecule 6	B3LYP/6-31G(d)							B3LYP-D3(BJ)/def2-TZVP
Neutral	GS	-846.8069	0.288475	-846.50284	0.055681	-846.55852		-847.19783
	TS	-846.76804	0.287776	-846.46522	0.054627	-846.51984	-116.97	-847.158761
Radical cation	GS	-846.55664	0.288201	-846.25259	0.056856	-846.30945		-846.936875
	TS	-846.52539	0.28804	-846.22221	0.05544	-846.27765	-80.03	-846.906087
**Molecule 6**	**M06-2X/6-31G(d)**							**M06-2X/def2-TZVP**
Neutral	GS	-846.45568	0.291516	-846.14867	0.05551	-846.20418		-846.753729
	TS	-846.41361	0.290869	-846.10779	0.054501	-846.16229	-112.85	-846.712522
Radical	GS	-846.18397	0.290127	-845.87798	0.056898	-845.93488		-846.479359

cation	TS	-846.15499	0.290629	-845.84929	0.055358	-845.90465	-76.33	-846.446811

Molecule 7	B3LYP/6-31G(d)							B3LYP-D3(BJ)/def2-TZVP
Neutral	GS	-1000.4429	0.33507	-1000.0896	0.060959	-1000.1505		-1000.907726
	TS	-1000.3837	0.333974	-1000.0322	0.059515	-1000.0917	-41.3	-1000.84784
Radical cation	GS	-1000.1967	0.333819	-999.844	0.062889	-999.90689		-1000.651435
	TS	-1000.1477	0.334062	-999.79591	0.06044	-999.85635	-28.18	-1000.602444
**Molecule 7**	**M06-2X/6-31G(d)**							**M06-2X/def2-TZVP**
Neutral	GS	-1000.0342	0.338669	-999.67738	0.060738	-999.73811		-1000.385148
	TS	-999.97213	0.337709	-999.61697	0.059318	-999.67629	-41.02	-1000.32362
Radical cation	GS	-999.77001	0.337508	-999.414	0.062057	-999.47606		-1000.113934
	TS	-999.71796	0.336872	-999.3634	0.060406	-999.42381	-32.43	-1000.06212

Dichloromethane in SMD

		E/au	ZPE/au	H/au	T.qh-S/au	qh-G(T)/au	imag. v	
**Molecule 1**	**B3LYP/6-31G(d)**							**B3LYP-D3(BJ)/def2-TZVP**
Neutral	GS	-770.611198	0.275593	-770.320446	0.055582	-770.376029		-770.966937
	TS	-770.57496	0.276131	-770.284746	0.052798	-770.337544	-45.86	-770.930832
Radical cation	GS	-770.414602	0.276134	-770.123431	0.055405	-770.178836		-770.761921
	TS	-770.389323	0.276352	-770.098831	0.053554	-770.152385	-11.31	-770.737209
**Molecule 1**	**M062X/6-31G(d)**							**M062X/def2-TZVP**
Neutral	GS	-770.284909	0.278464	-769.991438	0.05493	-770.046368		-770.559216
	TS	-770.247491	0.278901	-769.954609	0.052623	-770.007232	-40.79	-770.521473
Radical cation	GS	-770.070908	0.27812	-769.77785	0.055195	-769.833046		-770.338734
	TS	-770.046426	0.278442	-769.753885	0.053501	-769.807386	-33.27	-770.314355

Molecule 2	B3LYP/6-31G(d)							B3LYP-D3(BJ)/def2-TZVP
Neutral	GS	-921.053312	0.283727	-920.752202	0.059483	-920.811685		-921.494807
	TS	-920.992402	0.283289	-920.692888	0.056629	-920.749517	-14.31	-921.432553
Radical cation	GS	-920.856029	0.284342	-920.554499	0.059434	-920.613933		-921.288277
	TS	-920.815463	0.283872	-920.515163	0.057873	-920.573036	-41.89	-921.247037
**Molecule 2**	**M062X/6-31G(d)**							**M062X/def2-TZVP**
Neutral	GS	-920.683813	0.286513	-920.379882	0.059615	-920.439497		-921.03632
	TS	-920.623347	0.286844	-920.320513	0.056234	-920.376747	-15.72	-920.97387
Radical cation	GS	-920.471216	0.286775	-920.167334	0.059292	-920.226626		-920.815819
	TS	-920.43145	0.286849	-920.12829	0.057691	-920.185981	-52.22	-920.775211

Molecule 3	B3LYP/6-31G(d)							B3LYP-D3(BJ)/def2-TZVP
Neutral	GS	-999.65366	0.340679	-999.292416	0.066318	-999.358734		-1000.120856
	TS	-999.591247	0.340461	-999.231657	0.062532	-999.294189	-14.62	-1000.060237
Radical cation	GS	-999.465391	0.340534	-999.104095	0.066936	-999.171032		-999.923938
	TS	-999.426186	0.341267	-999.065601	0.063637	-999.129238	-27.15	-999.886602
**Molecule 3**	**M062X/6-31G(d)**							**M062X/def2-TZVP**

Neutral	GS	-999.239993	0.344724	-998.875045	0.065144	-998.940189		-999.610572
	TS	-999.181041	0.344406	-998.817753	0.062063	-998.879816	-17.00	-999.550071
Radical cation	GS	-999.036873	0.343863	-998.672619	0.066015	-998.738634		-999.399561
	TS	-999.002114	0.344925	-998.638085	0.063226	-998.701311	-35.75	-999.364435

Molecule 4		B3LYP/6-31G(d)						B3LYP-D3(BJ)/def2-TZVP
Neutral	GS	-960.353474	0.3123	-960.022186	0.062896	-960.085082		-960.807929
	TS	-960.291768	0.311895	-959.962146	0.059717	-960.021863	-30.27	-960.746256
Radical cation	GS	-960.161034	0.312589	-959.829568	0.062943	-959.892511		-960.606417
	TS	-960.120496	0.312403	-959.790139	0.060918	-959.851057	-36.81	-960.566364
**Molecule 4**		**M062X/6-31G(d)**						**M062X/def2-TZVP**
Neutral	GS	-959.962222	0.315708	-959.627661	0.062464	-959.690125		-960.32374
	TS	-959.902011	0.315753	-959.56881	0.059201	-959.628011	-24.02	-960.261851
Radical cation	GS	-959.754217	0.315315	-959.420162	0.062621	-959.482784		-960.107835
	TS	-959.715399	0.315291	-959.382135	0.061029	-959.443164	-72.23	-960.06841

Molecule 5		B3LYP/6-31G(d)						B3LYP-D3(BJ)/def2-TZVP
Neutral	GS	-881.326498	0.308795	-880.999763	0.060263	-881.060026		-881.750033
	TS	-881.25987	0.307895	-880.935037	0.057715	-880.992751	-9.18	-881.684064
Radical cation	GS	-881.149305	0.308681	-880.822462	0.061138	-880.8836		-881.564246
	TS	-881.108583	0.309265	-880.782339	0.058693	-880.841032	-38.05	-881.523619
**Molecule 5**		**M062X/6-31G(d)**						**M062X/def2-TZVP**
Neutral	GS	-880.962822	0.311338	-880.633522	0.060308	-880.693829		-881.29251
	TS	-880.896488	0.311194	-880.568552	0.057394	-880.625946	-9.93	-881.225575
Radical cation	GS	-880.772689	0.311736	-880.443109	0.060457	-880.503566		-881.094982
	TS	-880.732952	0.312351	-880.403757	0.058521	-880.462278	-59.30	-881.05448

Molecule 6		B3LYP/6-31G(d)						B3LYP-D3(BJ)/def2-TZVP
Neutral	GS	-846.832556	0.288455	-846.528506	0.055045	-846.583551		-847.222839
	TS	-846.795257	0.287633	-846.492509	0.054774	-846.547284	-153.57	-847.185347
Radical cation	GS	-846.635321	0.288248	-846.331301	0.056701	-846.388002		-847.017212
	TS	-846.605053	0.288198	-846.301741	0.055417	-846.357158	-107.96	-846.987289
**Molecule 6**		**M062X/6-31G(d)**						**M062X/def2-TZVP**
Neutral	GS	-846.483137	0.291436	-846.176216	0.054857	-846.231073		-846.78113
	TS	-846.442938	0.290852	-846.137115	0.054546	-846.191661	-136.86	-846.741764
Radical cation	GS	-846.268355	0.290474	-845.962159	0.056625	-846.018784		-846.560401
	TS	-846.236001	0.290575	-845.93037	0.055336	-845.985706	-103.00	-846.528909

Molecule 7		B3LYP/6-31G(d)						B3LYP-D3(BJ)/def2-TZVP
Neutral	GS	-1000.472325	0.335089	-1000.118928	0.060981	-1000.179909		-1000.93689
	TS	-1000.414761	0.333685	-1000.063398	0.059758	-1000.123156	-80.48	-1000.878575
Radical cation	GS	-1000.27625	0.333974	-999.923598	0.062411	-999.986009		-1000.732788
	TS	-1000.228103	0.333402	-999.876845	0.060745	-999.93759	-70.87	-1000.684456

Molecule 7	M062X/6-31G(d)							M062X/def2-TZVP
Neutral	GS	-1000.06577	0.338639	-999.708992	0.060677	-999.769669		-1000.416658
	TS	-<br>1000.005446	0.337419	-999.650521	0.05946	-999.709981	-81.14	-1000.356796
Radical cation	GS	-999.851487	0.337227	-999.495792	0.061973	-999.557764		-1000.196579
	TS	-999.799815	0.335724	-999.44615	0.060981	-999.507131	-77.84	-1000.145102

4. Cartesian Coordinates

Gas Phase

Molecule 1 (neutral TS b3lyp)
C -2.36787 1.45731 -0.34768
C -1.98464 0.11139 -0.06908
C -0.61766 -0.41468 -0.09673
C 0.61766 0.41468 0.09673
C 1.98464 -0.11139 0.06908
C 2.36787 -1.45731 0.34768
C 3.67454 -1.89080 0.25582
C 4.70499 -1.00512 -0.12002
C 4.40418 0.32640 -0.27818
C 3.07615 0.81082 -0.12851
C 2.85193 2.21029 -0.07324
C 1.60816 2.67513 0.26682
C 0.51991 1.78359 0.35126
C -0.51991 -1.78359 -0.35126
C -1.60816 -2.67513 -0.26682
C -2.85193 -2.21029 0.07324
C -3.07615 -0.81082 0.12851
C -4.40418 -0.32640 0.27818
C -4.70499 1.00512 0.12002
C -3.67454 1.89080 -0.25582
H -1.63700 2.15535 -0.72824
H -3.91074 2.92252 -0.50160
H -5.72709 1.35905 0.22291
H -5.19100 -1.05072 0.47723
H -3.68907 -2.88709 0.22216
H -1.43478 -3.73464 -0.43417
H 0.43280 -2.20681 -0.62422
H 1.63700 -2.15535 0.72824
H 3.91074 -2.92252 0.50160
H 5.72709 -1.35905 -0.22291
H 5.19100 1.05072 -0.47723
H 3.68907 2.88709 -0.22216
H 1.43478 3.73464 0.43417
H -0.43280 2.20681 0.62422

Molecule 1 (radical cation TS b3lyp)
C -2.35324 1.43971 -0.39794
C -1.96525 0.11442 -0.10146
C -0.59162 -0.40172 -0.13084
C 0.59162 0.40172 0.13084
C 1.96525 -0.11442 0.10146
C 2.35324 -1.43971 0.39794
C 3.67633 -1.85937 0.28000
C 4.68167 -0.97118 -0.12667
C 4.36098 0.36160 -0.31116
C 3.03009 0.82158 -0.14788
C 2.77544 2.21358 -0.09762
C 1.52629 2.68036 0.29252
C 0.46836 1.78750 0.42545
C -0.46836 -1.78750 -0.42545
C -1.52629 -2.68036 -0.29252
C -2.77544 -2.21358 0.09762
C -3.03009 -0.82158 0.14788
C -4.36098 -0.36160 0.31116
C -4.68167 0.97118 0.12667
C -3.67633 1.85937 -0.28000
H -1.63654 2.13689 -0.81132
H -3.92939 2.88475 -0.53196
H -5.70559 1.31283 0.23758
H -5.13574 -1.08691 0.54324
H -3.59418 -2.90658 0.27023
H -1.36660 -3.73739 -0.47841
H 0.48477 -2.17396 -0.75507
H 1.63654 -2.13689 0.81132
H 3.92939 -2.88475 0.53196
H 5.70559 -1.31283 -0.23758
H 5.13574 1.08691 -0.54324
H 3.59418 2.90658 -0.27023
H 1.36660 3.73739 0.47841
H -0.48477 2.17396 0.75507

Molecule 1 (radical cation TS m062x)
C -2.35140 1.45136 -0.35428
C -1.97437 0.10700 -0.06252
C -0.61445 -0.41837 -0.09659
C 0.61445 0.41837 0.09659
C 1.97437 -0.10700 0.06252
C 2.35140 -1.45136 0.35428
C 3.65203 -1.88425 0.26350
C 4.68194 -1.00224 -0.12339
C 4.38402 0.32358 -0.28687
C 3.05708 0.80675 -0.13647
C 2.83506 2.20640 -0.07639
C 1.60054 2.66858 0.27803
C 0.51180 1.77516 0.36460
C -0.51180 -1.77516 -0.36460
C -1.60054 -2.66858 -0.27803
C -2.83506 -2.20640 0.07639
C -3.05708 -0.80675 0.13647
C -4.38402 -0.32358 0.28687
C -4.68194 1.00224 0.12339
C -3.65203 1.88425 -0.26350
H -1.61740 2.14019 -0.74663
H -3.88923 2.91167 -0.52186
H -5.70187 1.35888 0.22488
H -5.16902 -1.04693 0.49160
H -3.67340 -2.88043 0.22869
H -1.42901 -3.72610 -0.45199
H 0.43995 -2.19373 -0.64907

H	1.61740	-2.14019	0.74663
H	3.88923	-2.91167	0.52186
H	5.70187	-1.35888	-0.22488
H	5.16902	1.04693	-0.49160
H	3.67340	2.88043	-0.22869
H	1.42901	3.72610	0.45199
H	-0.43995	2.19373	0.64907

Molecule 1 (radical cation TS m062x)

C	2.18815	-1.40142	-0.49703
C	1.92585	-0.07797	-0.08951
C	0.59165	0.51211	-0.00167
C	-0.58669	-0.28421	0.26151
C	-1.97443	0.16208	0.11395
C	-2.45280	1.47927	0.26773
C	-3.77813	1.80352	0.01923
C	-4.70038	0.82978	-0.38535
C	-4.29678	-0.48634	-0.42860
C	-2.96128	-0.84563	-0.13217
C	-2.64777	-2.21390	0.05711
C	-1.41436	-2.58755	0.56342
C	-0.41156	-1.63324	0.66976
C	0.52942	1.91762	-0.17885
C	1.64501	2.73222	-0.04059
C	2.88550	2.16632	0.21459
C	3.05726	0.76405	0.13724
C	4.36144	0.21756	0.15305
C	4.57240	-1.11220	-0.14687
C	3.48066	-1.90767	-0.51802
H	1.38308	-2.02197	-0.87218
H	3.64428	-2.92745	-0.85048
H	5.57557	-1.52369	-0.14945
H	5.19869	0.87541	0.36718
H	3.75519	2.79541	0.38184
H	1.53728	3.80816	-0.11754
H	-0.41169	2.38903	-0.41079
H	-1.82127	2.25012	0.68487
H	-4.10586	2.82710	0.16846
H	-5.72811	1.10098	-0.59909
H	-5.00813	-1.27609	-0.65198
H	-3.42452	-2.95517	-0.10871
H	-1.22095	-3.61404	0.85315
H	0.54753	-1.93413	1.07108

Molecule 2 (neutral TS b3lyp - anti)

C	2.36747	1.30469	-0.59078
C	1.98113	0.03037	-0.08535
C	0.58819	-0.44799	-0.07428
C	-0.58819	0.44799	0.07428
C	-1.98113	-0.03037	0.08535
C	-2.36747	-1.30469	0.59078
C	-3.68245	-1.73806	0.56779
C	-4.70200	-0.91814	0.05028
C	-4.38694	0.36970	-0.32916
C	-3.05909	0.85561	-0.25752
C	-2.79475	2.24776	-0.37492
C	-1.57723	2.71876	0.02258
C	-0.51004	1.83495	0.34480
C	3.68245	1.73806	-0.56779
C	4.70200	0.91814	-0.05028
C	4.38694	-0.36970	0.32916
C	3.05909	-0.85561	0.25752
C	2.79475	-2.24776	0.37492
C	1.57723	-2.71876	-0.02258
C	0.51004	-1.83495	-0.34480
O	-0.52916	-2.48893	-0.92758
O	0.52916	2.48893	0.92758
H	1.62570	1.93885	-1.05830
H	-1.62570	-1.93885	1.05830
H	-3.92559	-2.71315	0.98119
H	-5.72791	-1.27257	0.01052
H	-5.16856	1.05977	-0.63870
H	-3.59195	2.92595	-0.66649
H	-1.36976	3.78090	0.10730
H	3.92559	2.71315	-0.98119
H	5.72791	1.27257	-0.01052
H	5.16856	-1.05977	0.63870
H	3.59195	-2.92595	0.66649
H	1.36976	-3.78090	-0.10730
H	-1.26784	-1.88037	-1.09663
H	1.26784	1.88037	1.09663

Molecule 2 (neutral TS b3lyp - syn)

C	-0.55278	-1.35780	1.80031
C	-0.19145	-1.68506	0.46667
C	-0.17108	-0.73056	-0.65141
C	0.17108	0.73056	-0.65141
C	0.19145	1.68506	0.46667
C	0.55278	1.35780	1.80031
C	0.52601	2.28752	2.82375
C	0.14287	3.61977	2.57869
C	-0.08038	4.01054	1.27702
C	0.01016	3.08766	0.20461
C	0.08038	3.56665	-1.13287
C	0.48142	2.71470	-2.12058
C	0.60004	1.32127	-1.86540
C	-0.52601	-2.28752	2.82375
C	-0.14287	-3.61977	2.57869
C	0.08038	-4.01054	1.27702
C	-0.01016	-3.08766	0.20461
C	-0.08038	-3.56665	-1.13287
C	-0.48142	-2.71470	-2.12058
C	-0.60004	-1.32127	-1.86540
O	-1.11802	-0.64169	-2.92619
O	1.11802	0.64169	-2.92619
H	-0.93431	-0.37138	2.01588
H	0.93431	0.37138	2.01588
H	0.83269	1.98583	3.82194
H	0.09390	4.33948	3.39084
H	-0.26948	5.05474	1.03852
H	-0.07158	4.62403	-1.33191
H	0.70641	3.05506	-3.12642
H	-0.83269	-1.98583	3.82194
H	-0.09390	-4.33948	3.39084
H	0.26948	-5.05474	1.03852
H	0.07158	-4.62403	-1.33191
H	-0.70641	-3.05506	-3.12642
H	-1.24467	0.29112	-2.68403
H	1.24467	-0.29112	-2.68403

Molecule 2 (radical cation TS b3lyp - anti)

C	-2.67489	1.42286	0.00945
C	-2.02531	0.16733	0.08251
C	-0.61588	-0.08836	0.43402
C	0.59252	0.68054	0.12138
C	1.80885	-0.06231	-0.25147

C	1.75636	-1.34062	-0.85494
C	2.91382	-2.03173	-1.18951
C	4.17703	-1.47461	-0.94327
C	4.26383	-0.19897	-0.40750
C	3.09950	0.53179	-0.08704
C	3.18487	1.89662	0.31472
C	2.05419	2.66660	0.39273
C	0.77505	2.10387	0.18390
C	-3.97369	1.55940	-0.47300
C	-4.70263	0.44561	-0.90216
C	-4.15271	-0.81206	-0.71561
C	-2.85748	-0.97535	-0.18067
C	-2.43279	-2.28059	0.21500
C	-1.27692	-2.45567	0.92470
C	-0.39298	-1.36701	1.07738
O	0.65693	-1.61810	1.86242
O	-0.20554	3.01360	0.12883
H	-2.25413	2.28944	0.50066
H	0.79577	-1.78139	-1.09616
H	2.83392	-3.00621	-1.66132
H	5.07737	-2.02345	-1.19988
H	5.23255	0.26901	-0.25625
H	4.16214	2.34418	0.47191
H	2.09730	3.73460	0.57811
H	-4.43029	2.54450	-0.48012
H	-5.70350	0.55858	-1.30523
H	-4.73233	-1.70230	-0.94384
H	-3.09426	-3.12206	0.02955
H	-0.99833	-3.40685	1.36449
H	1.24892	-0.84726	1.92029
H	-0.97082	2.66116	-0.35606

Molecule 2 (radical cation TS b3lyp - syn)

C	-0.23695	-1.58462	1.92758
C	-0.13818	-1.76051	0.53176
C	-0.21541	-0.70371	-0.49064
C	0.21541	0.70371	-0.49064
C	0.13818	1.76051	0.53176
C	0.23695	1.58462	1.92758
C	0.05934	2.63483	2.82200
C	-0.23695	3.92502	2.36796
C	-0.23729	4.16196	1.00355
C	0.00129	3.12118	0.08048
C	0.22483	3.45485	-1.28718
C	0.69037	2.51372	-2.16703
C	0.76224	1.16896	-1.75353
C	-0.05934	-2.63483	2.82200
C	0.23695	-3.92502	2.36796
C	0.23729	-4.16196	1.00355
C	-0.00129	-3.12118	0.08048
C	-0.22483	-3.45485	-1.28718
C	-0.69037	-2.51372	-2.16703
C	-0.76224	-1.16896	-1.75353
O	-1.35461	-0.35442	-2.63428
O	1.35461	0.35442	-2.63428
H	-0.55891	-0.63838	2.32530
H	0.55891	0.63838	2.32530
H	0.17466	2.44628	3.88510
H	-0.40569	4.73499	3.06994
H	-0.37989	5.16896	0.62137
H	0.12213	4.49067	-1.59784
H	1.02706	2.76232	-3.16749
H	-0.17466	-2.44628	3.88510
H	0.40569	-4.73499	3.06994
H	0.37989	-5.16896	0.62137
H	-0.12213	-4.49067	-1.59784
H	-1.02706	-2.76232	-3.16749
H	-1.46249	0.54289	-2.27186
H	1.46249	-0.54289	-2.27186

Molecule 2 (neutral TS m062x - anti)

C	2.35008	1.29787	-0.58836
C	1.97328	0.02676	-0.07310
C	0.58688	-0.45155	-0.06537
C	-0.58688	0.45155	0.06537
C	-1.97328	-0.02676	0.07310
C	-2.35008	-1.29787	0.58836
C	-3.65819	-1.73058	0.57376
C	-4.68108	-0.91360	0.05818
C	-4.37175	0.36823	-0.32672
C	-3.04439	0.85139	-0.26458
C	-2.78092	2.24458	-0.38369
C	-1.57021	2.71303	0.01690
C	-0.50463	1.82476	0.34063
C	3.65819	1.73058	-0.57376
C	4.68108	0.91360	-0.05818
C	4.37175	-0.36823	0.32672
C	3.04439	-0.85139	0.26458
C	2.78092	-2.24458	0.38369
C	1.57021	-2.71303	-0.01690
C	0.50463	-1.82476	-0.34063
O	-0.53175	-2.47111	-0.92330
O	0.53175	2.47111	0.92330
H	1.59810	1.92813	-1.04583
H	-1.59810	-1.92813	1.04583
H	-3.89919	-2.70259	0.99263
H	-5.70546	-1.26974	0.02659
H	-5.15333	1.05750	-0.63591
H	-3.57923	2.92041	-0.67562
H	-1.35635	3.77330	0.10178
H	3.89919	2.70259	-0.99263
H	5.70546	1.26974	-0.02659
H	5.15333	-1.05750	0.63591
H	3.57923	-2.92041	0.67562
H	1.35635	-3.77330	-0.10178
H	-1.23269	-1.84727	-1.16611
H	1.23269	1.84727	1.16611

Molecule 2 (neutral TS m062x - syn)

C	-0.46385	-1.40814	1.82419
C	-0.15625	-1.70603	0.47168
C	-0.18076	-0.73027	-0.61901
C	0.18076	0.73027	-0.61901
C	0.15625	1.70603	0.47168
C	0.46385	1.40814	1.82419
C	0.38922	2.35606	2.82039
C	0.01641	3.68095	2.52835
C	-0.13843	4.04122	1.21331
C	-0.00238	3.09258	0.17163
C	0.13843	3.54194	-1.17164
C	0.57241	2.66763	-2.11595
C	0.65358	1.27606	-1.82319
C	-0.38922	-2.35606	2.82039
C	-0.01641	-3.68095	2.52835
C	0.13843	-4.04122	1.21331
C	0.00238	-3.09258	0.17163

C	-0.13843	-3.54194	-1.17164
C	-0.57241	-2.66763	-2.11595
C	-0.65358	-1.27606	-1.82319
O	-1.16163	-0.56285	-2.85586
O	1.16163	0.56285	-2.85586
H	-0.85772	-0.43391	2.07023
H	0.85772	0.43391	2.07023
H	0.65719	2.08013	3.83583
H	-0.06955	4.41784	3.32013
H	-0.31195	5.07876	0.93953
H	0.00633	4.59593	-1.39821
H	0.83744	2.97425	-3.12210
H	-0.65719	-2.08013	3.83583
H	0.06955	-4.41784	3.32013
H	0.31195	-5.07876	0.93953
H	-0.00633	-4.59593	-1.39821
H	-0.83744	-2.97425	-3.12210
H	-1.25177	0.36866	-2.59722
H	1.25177	-0.36866	-2.59722

Molecule 2 (radical cation TS m062x -anti)

C	-2.65034	1.44120	-0.14581
C	-2.01451	0.19830	0.06776
C	-0.61695	-0.02952	0.45323
C	0.59800	0.71082	0.12129
C	1.76956	-0.07406	-0.28303
C	1.64236	-1.35217	-0.86988
C	2.75800	-2.08665	-1.22889
C	4.04831	-1.57514	-1.02836
C	4.20179	-0.30619	-0.50707
C	3.07703	0.46551	-0.15224
C	3.22639	1.81991	0.26839
C	2.13339	2.62503	0.41421
C	0.82852	2.11195	0.23953
C	-3.93356	1.53318	-0.66139
C	-4.66143	0.38666	-0.98846
C	-4.12114	-0.84535	-0.67570
C	-2.83743	-0.95666	-0.11277
C	-2.41533	-2.23556	0.37326
C	-1.26069	-2.37279	1.07824
C	-0.37968	-1.27175	1.15420
O	0.68238	-1.47392	1.91585
O	-0.13114	3.03945	0.29331
H	-2.23644	2.35303	0.25525
H	0.65554	-1.75231	-1.07930
H	2.62897	-3.06153	-1.68700
H	4.91675	-2.16049	-1.30951
H	5.19090	0.12569	-0.38580
H	4.22577	2.22556	0.39730
H	2.21755	3.68318	0.63504
H	-4.38326	2.51358	-0.77823
H	-5.65488	0.46402	-1.41539
H	-4.69691	-1.75313	-0.83122
H	-3.08281	-3.08241	0.24221
H	-0.96872	-3.29450	1.56765
H	1.27525	-0.70231	1.92332
H	-0.85128	2.79718	-0.30669

Molecule 2 (radical cation TS m062x - syn)

C	-0.13208	-1.62651	1.94245
C	-0.11200	-1.77463	0.54331
C	-0.22108	-0.69852	-0.44901
C	0.22108	0.69852	-0.44901
C	0.11200	1.77463	0.54331
C	0.13208	1.62651	1.94245
C	-0.08851	2.69427	2.79725
C	-0.34937	3.97400	2.29849
C	-0.27487	4.17828	0.93551
C	-0.00030	3.11471	0.05535
C	0.27487	3.41764	-1.31321
C	0.75063	2.46019	-2.15820
C	0.79253	1.12433	-1.70774
C	0.08851	-2.69427	2.79725
C	0.34937	-3.97400	2.29849
C	0.27487	-4.17828	0.93551
C	0.00030	-3.11471	0.05535
C	-0.27487	-3.41764	-1.31321
C	-0.75063	-2.46019	-2.15820
C	-0.79253	-1.12433	-1.70774
O	-1.36670	-0.27811	-2.55443
O	1.36670	0.27811	-2.55443
H	-0.43667	-0.69223	2.37724
H	0.43667	0.69223	2.37724
H	-0.03394	2.53089	3.86847
H	-0.54865	4.80027	2.97157
H	-0.39096	5.17540	0.52084
H	0.18944	4.44862	-1.64442
H	1.10956	2.67486	-3.15809
H	0.03394	-2.53089	3.86847
H	0.54865	-4.80027	2.97157
H	0.39096	-5.17540	0.52084
H	-0.18944	-4.44862	-1.64442
H	-1.10956	-2.67486	-3.15809
H	-1.47156	0.60724	-2.16546
H	1.47156	-0.60724	-2.16546

Molecule 3 (neutral TS b3lyp)

C	2.47079	0.98930	0.75656
C	1.96978	-0.17244	0.10583
C	0.54435	-0.50243	0.02487
C	-0.54404	0.50286	-0.02419
C	-1.96927	0.17232	-0.10628
C	-2.46915	-0.98934	-0.75803
C	-3.81779	-1.27258	-0.81825
C	-4.76535	-0.41664	-0.21467
C	-4.33326	0.76928	0.33061
C	-2.95597	1.11802	0.33076
C	-2.55146	2.44012	0.63791
C	-1.28134	2.84091	0.30970
C	-0.31457	1.90046	-0.13184
C	3.81960	1.27208	0.81519
C	4.76622	0.41548	0.21110
C	4.33313	-0.77067	-0.33289
C	2.95570	-1.11888	-0.33141
C	2.55045	-2.44107	-0.63712
C	1.28040	-2.84111	-0.30771
C	0.31426	-1.89987	0.13351
O	-0.84578	-2.31855	0.70757
O	0.84541	2.32017	-0.70525
H	1.78158	1.63304	1.28683
H	-1.77907	-1.63269	-1.28769
H	-4.15447	-2.15385	-1.35880
H	-5.82307	-0.66429	-0.23730
H	-5.04625	1.49301	0.71942
H	-3.27699	3.15308	1.02065
H	-1.00767	3.88612	0.39189

H	4.15717	2.15349	1.35497
H	5.82405	0.66278	0.23242
H	5.04545	-1.49498	-0.72186
H	3.27548	-3.15466	-1.01967
H	1.00628	-3.88628	-0.38884
C	1.16746	3.70045	-0.74474
H	0.42479	4.27053	-1.31692
H	1.25725	4.13021	0.26192
H	2.13559	3.75749	-1.24501
C	-1.16898	-3.69855	0.74775
H	-1.25826	-4.12896	-0.25867
H	-0.42726	-4.26881	1.32098
H	-2.13761	-3.75444	1.24718

Molecule 3 (radical cation TS b3lyp)

C	2.37495	-0.97081	-0.89476
C	1.93528	0.16261	-0.18397
C	0.51070	0.51901	-0.01830
C	-0.53849	-0.44409	0.10430
C	-1.99015	-0.16779	0.12995
C	-2.58049	0.93898	0.76737
C	-3.95539	1.14311	0.73733
C	-4.80163	0.25790	0.05296
C	-4.26249	-0.88601	-0.50874
C	-2.87579	-1.14392	-0.42567
C	-2.37374	-2.43854	-0.74439
C	-1.11815	-2.82220	-0.32729
C	-0.24758	-1.86574	0.23662
C	3.72636	-1.26634	-1.02239
C	4.70124	-0.44802	-0.43025
C	4.30618	0.71450	0.20723
C	2.93861	1.06130	0.28948
C	2.55257	2.36740	0.70533
C	1.28138	2.83102	0.45146
C	0.29350	1.95667	-0.05137
O	-0.84505	2.40071	-0.57449
O	0.84294	-2.19902	0.91950
H	1.65200	-1.60117	-1.40084
H	-1.96733	1.61963	1.34304
H	-4.37751	1.99192	1.26741
H	-5.87049	0.44111	0.01110
H	-4.90543	-1.62856	-0.97327
H	-3.03850	-3.16654	-1.20093
H	-0.81256	-3.85857	-0.40228
H	4.02934	-2.13215	-1.60400
H	5.75367	-0.69904	-0.51515
H	5.04535	1.40239	0.60847
H	3.30292	3.03964	1.11203
H	1.04425	3.87534	0.61246
C	1.21706	-3.57738	1.07741
H	0.41428	-4.13983	1.56302
H	1.46554	-4.02585	0.11003
H	2.10004	-3.55950	1.71460
C	-1.12415	3.80859	-0.64787
H	-1.21404	4.24205	0.35353
H	-0.34542	4.32463	-1.21686
H	-2.07787	3.87942	-1.16918

Molecule 3 (neutral TS m062x)

C	2.59133	0.96481	0.63119
C	1.99083	-0.19111	0.05850
C	0.55816	-0.46186	0.08736
C	-0.52738	0.55360	0.02185
C	-1.92847	0.18524	-0.13928
C	-2.34646	-1.01025	-0.78837
C	-3.67150	-1.33622	-0.93001
C	-4.68283	-0.49070	-0.41966
C	-4.32453	0.71583	0.11826
C	-2.96086	1.10184	0.20730
C	-2.61529	2.43635	0.53109
C	-1.33855	2.86387	0.30463
C	-0.32132	1.94193	-0.05851
C	3.94590	1.18287	0.57722
C	4.80364	0.25786	-0.05811
C	4.27958	-0.92621	-0.50320
C	2.89222	-1.20257	-0.38468
C	2.41329	-2.52299	-0.57102
C	1.16119	-2.84305	-0.12923
C	0.27007	-1.82320	0.29813
O	-0.87509	-2.13848	0.94941
O	0.85681	2.39144	-0.54955
H	1.97405	1.64933	1.19660
H	-1.59588	-1.64805	-1.24287
H	-3.94465	-2.24285	-1.46248
H	-5.72764	-0.77076	-0.50792
H	-5.08018	1.42663	0.44313
H	-3.38738	3.13094	0.84959
H	-1.09223	3.91418	0.40398
H	4.36389	2.06235	1.05871
H	5.86880	0.45418	-0.12662
H	4.92429	-1.70458	-0.90353
H	3.08362	-3.28946	-0.94937
H	0.83642	-3.87644	-0.11600
C	1.23552	3.73604	-0.35815
H	0.60446	4.41658	-0.94063
H	1.19529	4.01989	0.70120
H	2.26536	3.80299	-0.71115
C	-1.36132	-3.46134	0.90484
H	-1.47096	-3.81608	-0.12801
H	-0.70748	-4.14580	1.45749
H	-2.34277	-3.43122	1.37829

Molecule 3 (radical cation TS m062x)

C	2.19612	-1.03275	-0.92924
C	1.87699	0.14848	-0.23094
C	0.49172	0.57539	0.03308
C	-0.57036	-0.37037	0.18810
C	-2.02716	-0.16121	0.09069
C	-2.73498	0.94063	0.60218
C	-4.10536	1.05583	0.43406
C	-4.83326	0.08408	-0.26376
C	-4.18344	-1.05590	-0.68875
C	-2.80194	-1.21882	-0.46537
C	-2.21859	-2.50957	-0.64201
C	-0.99313	-2.80433	-0.10597
C	-0.21961	-1.75469	0.43709
C	3.50877	-1.40441	-1.15249
C	4.56994	-0.61296	-0.68574
C	4.28882	0.58809	-0.07190
C	2.95349	1.00105	0.11880
C	2.67001	2.33288	0.53939
C	1.40695	2.84624	0.42220
C	0.33285	2.00818	0.04170
O	-0.84165	2.50087	-0.30138
O	0.84533	-1.96266	1.18713
H	1.39884	-1.64374	-1.34297

H	-2.22016	1.68606	1.18989
H	-4.62115	1.90608	0.86872
H	-5.90069	0.19971	-0.41535
H	-4.73574	-1.86713	-1.15416
H	-2.81767	-3.29623	-1.09181
H	-0.63130	-3.82439	-0.07964
H	3.71787	-2.30840	-1.71581
H	5.59648	-0.92067	-0.85101
H	5.08993	1.25377	0.23603
H	3.48910	2.97341	0.85368
H	1.22686	3.89693	0.60974
C	1.36822	-3.28173	1.33961
H	0.63185	-3.93203	1.81868
H	1.66394	-3.68639	0.36633
H	2.24288	-3.17273	1.97698
C	-1.08602	3.90681	-0.26867
H	-0.99455	4.29200	0.75098
H	-0.39681	4.42843	-0.93753
H	-2.10921	4.02424	-0.62112

Molecule 4 (neutral TS b3lyp)

C	-2.34464	-1.38989	0.70842
C	-1.99736	-0.15904	0.07574
C	-0.61334	0.31687	-0.04680
C	0.54126	-0.61151	-0.14919
C	1.96253	-0.22557	-0.11500
C	2.46509	1.00085	-0.62503
C	3.80624	1.32969	-0.56408
C	4.74136	0.44838	0.01323
C	4.31442	-0.80161	0.40272
C	2.95463	-1.18411	0.27956
C	2.59073	-2.55455	0.39269
C	1.36199	-2.94991	-0.05085
C	0.37299	-1.99392	-0.40788
C	-3.65296	-1.82133	0.80253
C	-4.70869	-1.05220	0.26926
C	-4.42794	0.18902	-0.25485
C	-3.09906	0.68468	-0.30186
C	-2.84552	2.04531	-0.59817
C	-1.60904	2.56526	-0.31295
C	-0.53120	1.72821	0.07855
O	0.59437	2.26540	0.61592
O	-0.70362	-2.57387	-1.00557
H	-1.56585	-1.98474	1.16821
H	1.79527	1.67297	-1.13988
H	4.14323	2.27002	-0.99356
H	5.78942	0.72527	0.08641
H	5.02646	-1.54466	0.75494
H	3.33023	-3.28203	0.71679
H	1.08780	-3.99631	-0.14096
H	-3.86764	-2.75556	1.31425
H	-5.73207	-1.41249	0.32287
H	-5.23026	0.84074	-0.59331
H	-3.65355	2.68626	-0.93999
H	-1.44493	3.63337	-0.39264
C	0.74453	3.67240	0.71883
H	0.75150	4.15840	-0.26586
H	-0.04554	4.11604	1.33718
H	1.71248	3.82378	1.19945
H	-1.37851	-1.90550	-1.20596

Molecule 4 (radical cation TS b3lyp)

C	2.79873	1.42933	0.20997
C	2.09052	0.21383	0.04440
C	0.66493	-0.02948	0.31492
C	-0.49491	0.83095	0.09445
C	-1.74036	0.21413	-0.38658
C	-1.74521	-0.95893	-1.17324
C	-2.93203	-1.53137	-1.61573
C	-4.16852	-0.95142	-1.29725
C	-4.19422	0.23496	-0.58117
C	-2.99741	0.84690	-0.15046
C	-3.01618	2.14448	0.43942
C	-1.84849	2.83873	0.62104
C	-0.60018	2.24316	0.33700
C	4.11703	1.57730	-0.20804
C	4.80747	0.51824	-0.81171
C	4.19513	-0.72060	-0.86610
C	2.87500	-0.90736	-0.39768
C	2.37104	-2.23150	-0.25211
C	1.19138	-2.47121	0.40680
C	0.36673	-1.37898	0.75505
O	-0.72149	-1.51015	1.50996
O	0.42763	3.10094	0.39124
H	2.39388	2.21865	0.82915
H	-0.80471	-1.40423	-1.47820
H	-2.89553	-2.42407	-2.23321
H	-5.09261	-1.40596	-1.63975
H	-5.13876	0.72841	-0.36929
H	-3.97045	2.61661	0.65548
H	-1.83871	3.87313	0.94770
H	4.62185	2.52194	-0.02899
H	5.82542	0.64953	-1.16326
H	4.73947	-1.58606	-1.23402
H	2.98778	-3.06310	-0.58126
H	0.89007	-3.48383	0.64344
C	-1.17803	-2.80838	1.92470
H	-1.44604	-3.41712	1.05527
H	-0.41684	-3.31195	2.52779
H	-2.06225	-2.61666	2.53086
H	1.18372	2.76179	-0.11740

Molecule 4 (neutral TS m062x)

C	2.29279	-1.40486	-0.69266
C	1.98678	-0.14933	-0.08953
C	0.62059	0.34596	0.06013
C	-0.53583	-0.58476	0.17689
C	-1.95536	-0.22705	0.08408
C	-2.49708	1.00150	0.54614
C	-3.83515	1.30031	0.42948
C	-4.73169	0.38338	-0.15306
C	-4.26997	-0.86661	-0.47872
C	-2.90896	-1.21350	-0.29805
C	-2.51875	-2.58220	-0.33673
C	-1.30621	-2.93182	0.16650
C	-0.34647	-1.93621	0.50397
C	3.58526	-1.85161	-0.81884
C	4.66856	-1.07878	-0.34733
C	4.42268	0.17449	0.14818
C	3.10124	0.68376	0.22776
C	2.87549	2.05280	0.50557
C	1.63956	2.58080	0.26360
C	0.54218	1.74363	-0.07011
O	-0.58999	2.27930	-0.57597
O	0.72960	-2.46025	1.13906
H	1.48636	-2.00379	-1.09805

H	-1.85726	1.69184	1.07616
H	-4.20589	2.24235	0.82339
H	-5.78027	0.63671	-0.27137
H	-4.95274	-1.63754	-0.82685
H	-3.23601	-3.33461	-0.65191
H	-1.00909	-3.96498	0.31182
H	3.77242	-2.80327	-1.30617
H	5.68368	-1.45385	-0.42661
H	5.24087	0.82596	0.44426
H	3.70589	2.68795	0.79972
H	1.48648	3.65136	0.32873
C	-0.83682	3.66298	-0.45626
H	-0.77162	3.99586	0.58723
H	-0.14192	4.24746	-1.06937
H	-1.85455	3.80951	-0.81983
H	1.35972	-1.76067	1.36783

Molecule 4 (radical cation TS m062x)

C	2.79909	1.46480	0.01020
C	2.09255	0.24060	0.04211
C	0.68070	0.03642	0.36563
C	-0.48809	0.86905	0.10050
C	-1.67337	0.20108	-0.43858
C	-1.57716	-0.98383	-1.19885
C	-2.70981	-1.61704	-1.68206
C	-3.98498	-1.08674	-1.43939
C	-4.10222	0.10416	-0.75049
C	-2.95929	0.77117	-0.26651
C	-3.06804	2.05779	0.33965
C	-1.94966	2.78797	0.62391
C	-0.66249	2.24850	0.40935
C	4.09755	1.55054	-0.46056
C	4.77506	0.41759	-0.92643
C	4.16299	-0.81033	-0.79968
C	2.85618	-0.92397	-0.28323
C	2.34468	-2.22580	-0.00519
C	1.15838	-2.40234	0.64671
C	0.34768	-1.27028	0.88299
O	-0.76879	-1.33108	1.58709
O	0.33421	3.11425	0.61843
H	2.42136	2.33753	0.51978
H	-0.59977	-1.38470	-1.44831
H	-2.60454	-2.51957	-2.27557
H	-4.86724	-1.58935	-1.82012
H	-5.07717	0.55606	-0.59187
H	-4.05419	2.48373	0.50128
H	-1.99951	3.80629	0.99262
H	4.60312	2.51021	-0.43340
H	5.78307	0.49805	-1.31706
H	4.69206	-1.72004	-1.06877
H	2.96393	-3.08462	-0.24834
H	0.83790	-3.38700	0.96139
C	-1.33916	-2.59666	1.92043
H	-1.53158	-3.16973	1.00774
H	-0.68283	-3.15071	2.59618
H	-2.27646	-2.36598	2.42191
H	1.06503	2.91838	0.01520

Molecule 5 (neutral TS b3lyp)

C	-2.37970	1.28446	0.61551
C	-1.98106	0.02919	0.07966
C	-0.58374	-0.43734	0.07706
C	0.58218	0.46014	-0.05548
C	1.97279	-0.02546	-0.08635
C	2.34123	-1.28188	-0.64110
C	3.65288	-1.72088	-0.65741
C	4.68734	-0.92873	-0.12246
C	4.38706	0.34343	0.31682
C	3.06044	0.83746	0.27843
C	2.80055	2.22475	0.44541
C	1.58940	2.71359	0.05008
C	0.50731	1.85719	-0.32775
C	-3.69807	1.70411	0.60135
C	-4.71011	0.89023	0.05694
C	-4.38211	-0.38292	-0.35947
C	-3.04923	-0.85595	-0.29089
C	-2.76735	-2.24230	-0.42921
C	-1.55695	-2.70752	-0.00419
C	-0.49317	-1.82854	0.37363
H	-1.64093	1.91819	1.08407
H	1.58310	-1.89906	-1.10160
H	3.88461	-2.68178	-1.10989
H	5.71168	-1.29099	-0.11395
H	5.17690	1.01662	0.64338
H	3.59573	2.89135	0.76878
H	1.40809	3.78619	0.02330
H	-3.95258	2.66614	1.03901
H	-5.73955	1.23630	0.02489
H	-5.15554	-1.07278	-0.69047
H	-3.54796	-2.92531	-0.75381
H	-1.36151	-3.77680	0.04510
N	-0.56409	2.50875	-0.92136
H	-0.31588	3.36091	-1.40640
H	-1.23175	1.94206	-1.42341
N	0.58214	-2.45215	0.98923
H	1.23725	-1.86576	1.48534
H	0.34387	-3.29969	1.48714

Molecule 5 (radical cation TS b3lyp)

C	-2.69930	1.39735	0.09241
C	-2.03111	0.15267	0.08937
C	-0.61129	-0.08460	0.41623
C	0.58157	0.69473	0.09821
C	1.80441	-0.05065	-0.26329
C	1.75139	-1.31614	-0.89194
C	2.90660	-2.01584	-1.21037
C	4.17191	-1.48278	-0.91697
C	4.25962	-0.21990	-0.35603
C	3.09558	0.51957	-0.05654
C	3.17882	1.88025	0.37030
C	2.06018	2.65886	0.39505
C	0.76169	2.12825	0.11389
C	-4.01636	1.52995	-0.33616
C	-4.73766	0.42232	-0.79525
C	-4.15474	-0.83114	-0.70179
C	-2.84246	-0.99238	-0.21488
C	-2.37355	-2.30930	0.09912
C	-1.22772	-2.48604	0.81167
C	-0.37720	-1.36864	1.06770
H	-2.23217	2.25308	0.55571
H	0.79023	-1.73766	-1.16446
H	2.82696	-2.97659	-1.71050
H	5.07145	-2.03866	-1.16166
H	5.22938	0.23406	-0.17061
H	4.15196	2.31498	0.58066
H	2.13686	3.72430	0.59648

H	-4.49236	2.50493	-0.28617
H	-5.75430	0.53361	-1.15788
H	-4.72306	-1.72000	-0.96224
H	-3.00536	-3.15926	-0.14284
H	-0.94366	-3.46293	1.19246
N	-0.22875	3.04133	-0.05533
H	0.04490	4.01550	-0.09620
H	-1.02234	2.80402	-0.63204
N	0.62592	-1.52880	1.94862
H	1.29368	-0.79165	2.12459
H	0.84543	-2.44467	2.31716

Molecule 5 (neutral TS m062x)

C	-2.41700	1.28522	0.56682
C	-1.98333	0.03178	0.05874
C	-0.58516	-0.40645	0.10589
C	0.57871	0.50043	-0.01365
C	1.94929	-0.01728	-0.08635
C	2.25783	-1.27536	-0.67113
C	3.54877	-1.74754	-0.73973
C	4.62301	-0.99290	-0.22934
C	4.37639	0.27450	0.23795
C	3.06384	0.80157	0.25387
C	2.84584	2.19108	0.45774
C	1.64017	2.71352	0.11338
C	0.52565	1.89098	-0.25127
C	-3.73631	1.67816	0.50776
C	-4.71716	0.83476	-0.04661
C	-4.35625	-0.43724	-0.41993
C	-3.01775	-0.87801	-0.30779
C	-2.70835	-2.26382	-0.39030
C	-1.50689	-2.68784	0.07998
C	-0.46772	-1.77167	0.44478
H	-1.70101	1.93659	1.04700
H	1.46067	-1.86200	-1.10811
H	3.73802	-2.70627	-1.21297
H	5.63469	-1.38445	-0.26243
H	5.19296	0.92045	0.55102
H	3.66719	2.82903	0.77136
H	1.47686	3.78844	0.12242
H	-4.02118	2.64040	0.92261
H	-5.75043	1.16003	-0.11254
H	-5.10538	-1.15177	-0.75218
H	-3.46907	-2.96996	-0.71061
H	-1.28443	-3.74857	0.16537
N	-0.53917	2.59166	-0.80052
H	-0.25064	3.42243	-1.30042
H	-1.21822	2.04641	-1.31329
N	0.61489	-2.35843	1.08227
H	1.25391	-1.73337	1.55435
H	0.36442	-3.16327	1.64125

Molecule 5 (radical cation TS m062x)

C	-2.68460	1.42118	-0.06131
C	-2.01991	0.18540	0.08025
C	-0.61394	-0.01593	0.45243
C	0.58874	0.73201	0.10671
C	1.75831	-0.06541	-0.29404
C	1.61582	-1.33391	-0.89815
C	2.71997	-2.08636	-1.25002
C	4.01873	-1.60586	-1.02051
C	4.18737	-0.34708	-0.48300
C	3.07156	0.44212	-0.14039
C	3.23296	1.79376	0.29676
C	2.15629	2.61411	0.39796
C	0.82333	2.14248	0.17131
C	-3.98054	1.50595	-0.54421
C	-4.68487	0.35758	-0.91546
C	-4.10633	-0.87491	-0.68197
C	-2.81226	-0.98050	-0.14592
C	-2.34523	-2.27127	0.27668
C	-1.20848	-2.39605	0.99935
C	-0.36494	-1.25365	1.17067
H	-2.24508	2.31529	0.34949
H	0.62315	-1.71023	-1.12424
H	2.57827	-3.04987	-1.72874
H	4.87960	-2.20493	-1.29636
H	5.18233	0.06520	-0.34110
H	4.23378	2.18121	0.46485
H	2.28543	3.66874	0.62389
H	-4.45834	2.47827	-0.60612
H	-5.68856	0.42969	-1.31905
H	-4.66235	-1.78784	-0.87529
H	-2.97606	-3.13548	0.08953
H	-0.91074	-3.34183	1.44087
N	-0.14549	3.08979	0.11923
H	0.16563	4.05316	0.14920
H	-0.92089	2.93245	-0.50697
N	0.66125	-1.35414	2.01840
H	1.30834	-0.58875	2.15408
H	0.88337	-2.23661	2.45970

Molecule 6 (neutral TS b3lyp)

C	-0.67954	3.22035	-0.49575
C	0.67953	3.22035	-0.49576
C	1.38247	2.01234	-0.26545
C	0.73639	0.73551	-0.34423
C	-0.73639	0.73551	-0.34423
C	-1.38248	2.01234	-0.26544
C	2.72691	2.16144	0.20614
C	1.66394	-0.40824	-0.20779
C	2.91427	-0.21404	0.47501
C	3.41609	1.10336	0.69496
C	3.73961	-1.31462	0.79876
H	4.64852	-1.12049	1.36257
C	3.44843	-2.58822	0.35717
C	2.35181	-2.75500	-0.49928
C	1.50715	-1.69299	-0.77483
H	3.13075	3.16822	0.26606
H	-1.24153	4.14833	-0.55285
H	1.24152	4.14833	-0.55286
H	4.38623	1.22504	1.16858
H	4.09597	-3.42459	0.60313
H	2.17042	-3.71323	-0.97763
H	-0.77122	-1.84256	-1.53803
C	-2.72691	2.16144	0.20615
H	-3.13075	3.16821	0.26608
C	-3.41610	1.10335	0.69497
H	-4.38623	1.22504	1.16858
C	-1.66394	-0.40824	-0.20779
C	-2.91427	-0.21404	0.47501
C	-3.73961	-1.31462	0.79875
H	-4.64853	-1.12050	1.36256
C	-3.44842	-2.58822	0.35716
H	-4.09596	-3.42460	0.60312
C	-2.35180	-2.75500	-0.49929

H	-2.17041	-3.71323	-0.97764
C	-1.50715	-1.69299	-0.77483
H	0.77123	-1.84257	-1.53803

Molecule 6 (radical cation TS b3lyp)

C	-0.67699	3.24700	-0.55427
C	0.67700	3.24700	-0.55428
C	1.37595	2.03230	-0.27531
C	0.72120	0.73776	-0.36689
C	-0.72120	0.73776	-0.36688
C	-1.37594	2.03231	-0.27531
C	2.68008	2.16509	0.24542
C	1.64192	-0.41549	-0.22390
C	2.86011	-0.23057	0.52016
C	3.34854	1.07801	0.76175
C	3.68712	-1.33198	0.84829
H	4.56958	-1.15072	1.45560
C	3.42156	-2.60005	0.35668
C	2.36239	-2.74700	-0.53941
C	1.51056	-1.67421	-0.83018
H	3.10778	3.15979	0.32450
H	-1.24447	4.16834	-0.64186
H	1.24448	4.16834	-0.64187
H	4.29725	1.20494	1.27542
H	4.06393	-3.43860	0.60430
H	2.19981	-3.69608	-1.04133
H	-0.80902	-1.81303	-1.63068
C	-2.68007	2.16510	0.24544
H	-3.10776	3.15980	0.32453
C	-3.34853	1.07801	0.76177
H	-4.29723	1.20495	1.27544
C	-1.64193	-0.41548	-0.22390
C	-2.86010	-0.23056	0.52016
C	-3.68712	-1.33197	0.84830
H	-4.56957	-1.15072	1.45561
C	-3.42157	-2.60004	0.35667
H	-4.06395	-3.43859	0.60429
C	-2.36241	-2.74699	-0.53943
H	-2.19983	-3.69606	-1.04136
C	-1.51058	-1.67420	-0.83019
H	0.80900	-1.81304	-1.63065

Molecule 6 (neutral TS m062x)

C	-0.67774	3.21425	-0.49426
C	0.67783	3.21421	-0.49441
C	1.37803	2.00380	-0.27176
C	0.73559	0.73632	-0.35066
C	-0.73558	0.73637	-0.35058
C	-1.37796	2.00387	-0.27152
C	2.72452	2.15636	0.20422
C	1.66246	-0.40904	-0.20742
C	2.90044	-0.21551	0.47845
C	3.40533	1.10428	0.70120
C	3.72801	-1.31119	0.80387
H	4.63239	-1.11405	1.37346
C	3.44300	-2.58182	0.36152
C	2.35356	-2.74889	-0.50170
C	1.51029	-1.69129	-0.77955
H	3.12610	3.16395	0.26201
H	-1.24124	4.14124	-0.54878
H	1.24137	4.14117	-0.54903
H	4.37287	1.22311	1.18033
H	4.09101	-3.41644	0.60876
H	2.18023	-3.70511	-0.98535
H	-0.78446	-1.83842	-1.55357
C	-2.72440	2.15643	0.20460
H	-3.12593	3.16402	0.26252
C	-3.40525	1.10433	0.70147
H	-4.37276	1.22316	1.18065
C	-1.66250	-0.40895	-0.20740
C	-2.90046	-0.21546	0.47851
C	-3.72806	-1.31117	0.80375
H	-4.63244	-1.11411	1.37338
C	-3.44310	-2.58174	0.36119
H	-4.09116	-3.41637	0.60828
C	-2.35365	-2.74875	-0.50203
H	-2.18036	-3.70492	-0.98580
C	-1.51036	-1.69113	-0.77972
H	0.78451	-1.83876	-1.55349

Molecule 6 (radical cation TS m062x)

C	-0.67434	3.24814	-0.57011
C	0.67434	3.24814	-0.57012
C	1.36926	2.02990	-0.28575
C	0.71518	0.74281	-0.39021
C	-0.71518	0.74281	-0.39021
C	-1.36926	2.02990	-0.28575
C	2.65842	2.15843	0.26214
C	1.62333	-0.41483	-0.23703
C	2.81244	-0.23963	0.53781
C	3.30120	1.06706	0.79545
C	3.62971	-1.33910	0.87467
H	4.49824	-1.16385	1.50296
C	3.37175	-2.59836	0.36610
C	2.33798	-2.73334	-0.55821
C	1.49808	-1.66052	-0.86086
H	3.08797	3.15073	0.35383
H	-1.24609	4.16632	-0.65862
H	1.24609	4.16632	-0.65863
H	4.23543	1.18444	1.33695
H	4.00556	-3.44067	0.61911
H	2.19040	-3.67616	-1.07473
H	-0.81972	-1.78424	-1.68347
C	-2.65841	2.15843	0.26214
H	-3.08797	3.15074	0.35384
C	-3.30120	1.06706	0.79545
H	-4.23543	1.18444	1.33695
C	-1.62333	-0.41483	-0.23703
C	-2.81244	-0.23962	0.53781
C	-3.62971	-1.33910	0.87467
H	-4.49825	-1.16385	1.50295
C	-3.37175	-2.59836	0.36609
H	-4.00556	-3.44066	0.61910
C	-2.33798	-2.73333	-0.55821
H	-2.19040	-3.67615	-1.07473
C	-1.49808	-1.66052	-0.86086
H	0.81972	-1.78424	-1.68347

Molecule 7 (neutral TS b3lyp)

C	1.19903	3.53612	-0.77084
C	2.31110	3.03341	-0.17089
C	2.34410	1.66855	0.22270
C	1.32290	0.76611	-0.19682
C	-0.00001	1.34980	-0.51012
C	-0.00002	2.76490	-0.77213
C	3.40532	1.22846	1.08247

C	1.75167	-0.63564	-0.17304
C	2.75147	-1.05721	0.76493
C	3.51498	-0.07134	1.46493
C	3.11899	-2.42128	0.83815
H	3.83573	-2.72302	1.59837
C	2.66301	-3.33228	-0.09441
C	1.86861	-2.87472	-1.15932
C	1.43024	-1.56232	-1.18780
H	4.08553	1.98412	1.46771
H	1.14733	4.57883	-1.07236
H	3.17591	3.65949	0.03350
H	4.26678	-0.39015	2.18220
H	2.97729	-4.37142	-0.04880
H	1.61056	-3.54543	-1.97431
C	-1.19908	3.53610	-0.77085
H	-1.14740	4.57881	-1.07237
C	-2.31115	3.03337	-0.17090
H	-3.17598	3.65944	0.03348
C	-1.32292	0.76609	-0.19682
C	-2.34413	1.66851	0.22269
C	-3.40535	1.22841	1.08246
H	-4.08557	1.98407	1.46769
C	-3.51499	-0.07139	1.46491
H	-4.26679	-0.39021	2.18219
C	-2.75145	-1.05725	0.76493
C	-1.75165	-0.63566	-0.17304
H	0.90692	-1.20215	-2.05985
C	-3.11895	-2.42133	0.83815
H	-3.83569	-2.72307	1.59837
C	-1.43020	-1.56235	-1.18779
H	-0.90689	-1.20218	-2.05984
C	-2.66295	-3.33232	-0.09440
H	-2.97721	-4.37147	-0.04878
C	-1.86855	-2.87476	-1.15931
H	-1.61048	-3.54547	-1.97429

Molecule 7 (radical cation TS b3lyp)

C	1.20064	3.53153	-0.78792
C	2.31532	3.02672	-0.16935
C	2.33708	1.67858	0.24668
C	1.30522	0.76934	-0.20642
C	0.00000	1.35993	-0.52889
C	0.00000	2.77020	-0.79307
C	3.36501	1.22205	1.12782
C	1.71139	-0.62501	-0.19044
C	2.68529	-1.06105	0.76843
C	3.44530	-0.08811	1.49481
C	3.02529	-2.42380	0.84390
H	3.71755	-2.74747	1.61625
C	2.56165	-3.33571	-0.10348
C	1.79621	-2.87400	-1.17416
C	1.38023	-1.54196	-1.21313
H	4.05258	1.95904	1.53205
H	1.16691	4.56834	-1.10903
H	3.17402	3.65847	0.03757
H	4.17514	-0.42296	2.22596
H	2.86266	-4.37722	-0.04909
H	1.54389	-3.53889	-1.99422
C	-1.20064	3.53153	-0.78792
H	-1.16692	4.56834	-1.10903
C	-2.31532	3.02672	-0.16935
H	-3.17402	3.65846	0.03757
C	-1.30522	0.76934	-0.20642
C	-2.33708	1.67857	0.24668
C	-3.36501	1.22205	1.12782
H	-4.05258	1.95904	1.53205
C	-3.44530	-0.08811	1.49481
H	-4.17514	-0.42296	2.22596
C	-2.68529	-1.06105	0.76843
C	-1.71139	-0.62501	-0.19044
H	0.92435	-1.16112	-2.11398
C	-3.02529	-2.42380	0.84390
H	-3.71755	-2.74747	1.61625
C	-1.38023	-1.54196	-1.21313
H	-0.92434	-1.16112	-2.11398
C	-2.56164	-3.33571	-0.10348
H	-2.86266	-4.37722	-0.04909
C	-1.79621	-2.87401	-1.17416
H	-1.54389	-3.53890	-1.99422

Molecule 7 (neutral TS m062x)

C	1.19907	3.53961	-0.75208
C	2.30483	3.02930	-0.15790
C	2.33216	1.65883	0.21611
C	1.31533	0.77328	-0.20719
C	0.00062	1.36063	-0.52406
C	0.00111	2.76688	-0.76780
C	3.38968	1.20788	1.07878
C	1.72330	-0.63193	-0.17648
C	2.70106	-1.06285	0.76339
C	3.47766	-0.08558	1.46601
C	3.05011	-2.42820	0.84000
H	3.75589	-2.73847	1.60604
C	2.58570	-3.33035	-0.09097
C	1.81220	-2.86165	-1.16428
C	1.40255	-1.54537	-1.19904
H	4.07279	1.95851	1.46695
H	1.14623	4.58542	-1.04013
H	3.17166	3.64814	0.05662
H	4.21937	-0.41586	2.18757
H	2.88157	-4.37349	-0.04143
H	1.55305	-3.52747	-1.98179
C	-1.19636	3.54040	-0.75248
H	-1.14283	4.58608	-1.04086
C	-2.30248	3.03094	-0.15826
H	-3.16896	3.65034	0.05601
C	-1.31456	0.77419	-0.20724
C	-2.33068	1.66056	0.21609
C	-3.38839	1.21055	1.07900
H	-4.07081	1.96177	1.46724
C	-3.47728	-0.08280	1.46640
H	-4.21907	-0.41243	2.18817
C	-2.70166	-1.06074	0.76365
C	-1.72371	-0.63070	-0.17644
H	0.90376	-1.16711	-2.07810
C	-3.05193	-2.42578	0.84036
H	-3.75781	-2.73539	1.60657
C	-1.40416	-1.54443	-1.19912
H	-0.90563	-1.16654	-2.07847
C	-2.58861	-3.32835	-0.09073
H	-2.88543	-4.37122	-0.04112
C	-1.81506	-2.86032	-1.16430
H	-1.55693	-3.52632	-1.98198

Molecule 7 (radical cation TS m062x)

C	1.19677	3.53011	-0.77944

C	2.30551	3.01903	-0.15824
C	2.31949	1.67104	0.24514
C	1.29390	0.77277	-0.21953
C	-0.00019	1.36444	-0.55098
C	-0.00034	2.76815	-0.80152
C	3.33825	1.20621	1.13824
C	1.68412	-0.62134	-0.19836
C	2.63266	-1.06452	0.76998
C	3.39660	-0.09676	1.50732
C	2.95718	-2.42419	0.84896
H	3.63831	-2.75569	1.62733
C	2.49080	-3.32778	-0.10189
C	1.74947	-2.85873	-1.18280
C	1.35760	-1.52436	-1.22907
H	4.02229	1.94058	1.55163
H	1.16391	4.56997	-1.08990
H	3.16328	3.64659	0.06252
H	4.11038	-0.44244	2.24877
H	2.77665	-4.37253	-0.04356
H	1.49966	-3.52089	-2.00514
C	-1.19754	3.52988	-0.77912
H	-1.16491	4.56981	-1.08940
C	-2.30613	3.01851	-0.15783
H	-3.16398	3.64592	0.06308
C	-1.29418	0.77247	-0.21948
C	-2.31990	1.67048	0.24534
C	-3.33879	1.20531	1.13815
H	-4.02303	1.93951	1.55152
C	-3.39700	-0.09772	1.50697
H	-4.11090	-0.44368	2.24817
C	-2.63271	-1.06520	0.76964
C	-1.68408	-0.62170	-0.19847
H	0.92153	-1.12936	-2.13422
C	-2.95668	-2.42501	0.84860
H	-3.63786	-2.75675	1.62682
C	-1.35695	-1.52466	-1.22908
H	-0.92095	-1.12946	-2.13417
C	-2.48971	-3.32844	-0.10209
H	-2.77517	-4.37330	-0.04379
C	-1.74832	-2.85915	-1.18287
H	-1.49813	-3.52124	-2.00515

Dichloromethane in SMD

Molecule 1 (neutral TS b3lyp)

C	-2.37199	1.46055	-0.34427
C	-1.98738	0.11332	-0.06965
C	-0.61916	-0.41465	-0.09604
C	0.61916	0.41465	0.09604
C	1.98738	-0.11332	0.06965
C	2.37199	-1.46055	0.34427
C	3.68068	-1.89216	0.25201
C	4.71152	-1.00422	-0.12020
C	4.40989	0.32852	-0.27475
C	3.07975	0.81067	-0.12545
C	2.85703	2.21132	-0.07058
C	1.61128	2.67682	0.26340
C	0.52228	1.78480	0.34587
C	-0.52228	-1.78480	-0.34587
C	-1.61128	-2.67682	-0.26340
C	-2.85703	-2.21132	0.07058
C	-3.07975	-0.81067	0.12545
C	-4.40989	-0.32852	0.27475
C	-4.71152	1.00422	0.12020
C	-3.68068	1.89216	-0.25201
H	-1.64409	2.16517	-0.71811
H	-3.91629	2.92513	-0.49427
H	-5.73392	1.35783	0.22159
H	-5.19572	-1.05448	0.46988
H	-3.69567	-2.88655	0.21928
H	-1.43604	-3.73679	-0.42570
H	0.42934	-2.21576	-0.60881
H	1.64409	-2.16517	0.71811
H	3.91629	-2.92513	0.49427
H	5.73392	-1.35783	-0.22159
H	5.19572	1.05448	-0.46988
H	3.69567	2.88655	-0.21928
H	1.43604	3.73679	0.42570
H	-0.42934	2.21576	0.60881

Molecule 1 (radical cation TS b3lyp)

C	-2.35319	1.44296	-0.39378
C	-1.96566	0.11664	-0.10094
C	-0.59179	-0.40053	-0.13191
C	0.59179	0.40053	0.13191
C	1.96566	-0.11664	0.10094
C	2.35319	-1.44296	0.39378
C	3.67688	-1.86053	0.27690
C	4.68292	-0.96983	-0.12284
C	4.36192	0.36340	-0.30582
C	3.03004	0.82026	-0.14616
C	2.77533	2.21360	-0.10107
C	1.52682	2.67942	0.28763
C	0.46952	1.78602	0.42680
C	-0.46952	-1.78602	-0.42680
C	-1.52682	-2.67942	-0.28763
C	-2.77533	-2.21360	0.10107
C	-3.03004	-0.82026	0.14616
C	-4.36192	-0.36340	0.30582
C	-4.68292	0.96983	0.12284
C	-3.67688	1.86053	-0.27690
H	-1.63789	2.14679	-0.79788
H	-3.92824	2.88774	-0.52391
H	-5.70811	1.31012	0.23047
H	-5.13441	-1.09271	0.53302
H	-3.59501	-2.90397	0.27826
H	-1.36345	-3.73702	-0.46705
H	0.48067	-2.17782	-0.75745
H	1.63789	-2.14679	0.79788
H	3.92824	-2.88774	0.52391
H	5.70811	-1.31012	-0.23047
H	5.13441	1.09271	-0.53302
H	3.59501	2.90397	-0.27826
H	1.36345	3.73702	0.46705
H	-0.48067	2.17782	0.75745

Molecule 1 (neutral TS m062x)

C	-2.35588	1.45514	-0.35080
C	-1.97726	0.10925	-0.06361
C	-0.61596	-0.41813	-0.09621

C	0.61596	0.41813	0.09621
C	1.97726	-0.10925	0.06361
C	2.35588	-1.45514	0.35080
C	3.65840	-1.88596	0.26007
C	4.68889	-1.00155	-0.12266
C	4.39017	0.32566	-0.28268
C	3.06097	0.80641	-0.13294
C	2.84066	2.20746	-0.07401
C	1.60396	2.67064	0.27387
C	0.51425	1.77681	0.35841
C	-0.51425	-1.77681	-0.35841
C	-1.60396	-2.67064	-0.27387
C	-2.84066	-2.20746	0.07401
C	-3.06097	-0.80641	0.13294
C	-4.39017	-0.32566	0.28268
C	-4.68889	1.00155	0.12266
C	-3.65840	1.88596	-0.26007
H	-1.62536	2.15173	-0.73585
H	-3.89562	2.91485	-0.51346
H	-5.70940	1.35752	0.22392
H	-5.17393	-1.05145	0.48412
H	-3.68036	-2.87993	0.22629
H	-1.42991	-3.72859	-0.44348
H	0.43643	-2.20359	-0.63369
H	1.62536	-2.15173	0.73585
H	3.89562	-2.91485	0.51346
H	5.70940	-1.35752	-0.22392
H	5.17393	1.05145	-0.48412
H	3.68036	2.87993	-0.22629
H	1.42991	3.72859	0.44348
H	-0.43643	2.20359	0.63369

Molecule 1 (radical cation TS m062x)

C	-2.33558	1.44313	-0.39874
C	-1.95465	0.11936	-0.09925
C	-0.58599	-0.39869	-0.13455
C	0.58599	0.39869	0.13455
C	1.95465	-0.11936	0.09925
C	2.33558	-1.44313	0.39874
C	3.65518	-1.85641	0.28377
C	4.65945	-0.96745	-0.12193
C	4.33981	0.36059	-0.31101
C	3.00931	0.81160	-0.15372
C	2.75316	2.20534	-0.10796
C	1.51291	2.67219	0.29447
C	0.45954	1.77875	0.44074
C	-0.45954	-1.77875	-0.44074
C	-1.51291	-2.67219	-0.29447
C	-2.75316	-2.20534	0.10796
C	-3.00931	-0.81160	0.15372
C	-4.33981	-0.36059	0.31101
C	-4.65945	0.96745	0.12193
C	-3.65518	1.85641	-0.28377
H	-1.61699	2.14347	-0.80500
H	-3.90991	2.88048	-0.53736
H	-5.68382	1.30850	0.22690
H	-5.10968	-1.09138	0.54151
H	-3.57275	-2.89340	0.29395
H	-1.35275	-3.72846	-0.47875
H	0.48930	-2.16348	-0.78607
H	1.61699	-2.14347	0.80500
H	3.90991	-2.88048	0.53736
H	5.68382	-1.30850	-0.22690
H	5.10968	1.09138	-0.54151
H	3.57275	2.89340	-0.29395
H	1.35275	3.72846	0.47875
H	-0.48930	2.16348	0.78607

Molecule 2 (neutral TS b3lyp)

C	2.36875	1.30735	-0.59189
C	1.98268	0.03198	-0.08847
C	0.58899	-0.44742	-0.07580
C	-0.58899	0.44742	0.07580
C	-1.98268	-0.03198	0.08847
C	-2.36875	-1.30735	0.59189
C	-3.68508	-1.73795	0.57262
C	-4.70581	-0.91553	0.05833
C	-4.39062	0.37178	-0.32525
C	-3.05997	0.85440	-0.25804
C	-2.79550	2.24700	-0.38100
C	-1.57712	2.71915	0.01577
C	-0.51173	1.83505	0.34378
C	3.68508	1.73795	-0.57262
C	4.70581	0.91553	-0.05833
C	4.39062	-0.37178	0.32525
C	3.05997	-0.85440	0.25804
C	2.79550	-2.24700	0.38100
C	1.57712	-2.71915	-0.01577
C	0.51173	-1.83505	-0.34378
O	-0.52720	-2.48817	-0.92922
O	0.52720	2.48817	0.92922
H	1.62754	1.94578	-1.05455
H	-1.62754	-1.94578	1.05455
H	-3.92939	-2.71330	0.98524
H	-5.73257	-1.26859	0.02314
H	-5.17099	1.06339	-0.63458
H	-3.59251	2.92395	-0.67545
H	-1.37342	3.78298	0.09565
H	3.92939	2.71330	-0.98524
H	5.73257	1.26859	-0.02314
H	5.17099	-1.06339	0.63458
H	3.59251	-2.92395	0.67545
H	1.37342	-3.78298	-0.09565
H	-1.24360	-1.86947	-1.15465
H	1.24360	1.86947	1.15465

Molecule 2 (radical cation TS b3lyp)

C	-2.67788	1.41538	0.03662
C	-2.02792	0.16084	0.07999
C	-0.61386	-0.09430	0.41366
C	0.58678	0.67817	0.09845
C	1.81323	-0.05831	-0.25449
C	1.77803	-1.33783	-0.85575
C	2.94615	-2.02071	-1.16948
C	4.20224	-1.45444	-0.90378
C	4.27207	-0.17889	-0.36694
C	3.09650	0.54311	-0.06677
C	3.16782	1.90670	0.34020
C	2.03100	2.67013	0.39081
C	0.76042	2.10234	0.15057
C	-3.98784	1.55404	-0.41375
C	-4.72428	0.44365	-0.83944
C	-4.16526	-0.81573	-0.69188
C	-2.85770	-0.98150	-0.18955
C	-2.42037	-2.29280	0.17290
C	-1.25908	-2.47160	0.87252

C	-0.38632	-1.37750	1.04733
O	0.65769	-1.62261	1.84269
O	-0.22269	3.01002	0.05810
H	-2.23179	2.28372	0.49886
H	0.82552	-1.78861	-1.11048
H	2.87974	-2.99705	-1.64026
H	5.11033	-1.99851	-1.14477
H	5.23279	0.29931	-0.19688
H	4.13980	2.35731	0.51935
H	2.06932	3.73868	0.57598
H	-4.44303	2.53985	-0.40303
H	-5.73548	0.55944	-1.21624
H	-4.74242	-1.70639	-0.92463
H	-3.07774	-3.13389	-0.02774
H	-0.97319	-3.43122	1.28965
H	1.21984	-0.83051	1.94970
H	-0.95607	2.66421	-0.48325

Molecule 2 (neutral TS m062x)

C	-2.36748	1.30727	0.57221
C	-1.97824	0.03333	0.07260
C	-0.58895	-0.43891	0.07942
C	0.58770	0.46084	-0.05791
C	1.97061	-0.02980	-0.07933
C	2.33105	-1.30647	-0.59398
C	3.63684	-1.74756	-0.59621
C	4.67311	-0.93686	-0.09389
C	4.37864	0.34685	0.29884
C	3.05211	0.83845	0.25373
C	2.80157	2.23361	0.38649
C	1.58955	2.71477	0.00169
C	0.51522	1.83710	-0.32305
C	-3.67956	1.72987	0.54392
C	-4.69346	0.90045	0.02763
C	-4.37275	-0.38324	-0.34495
C	-3.04069	-0.85507	-0.26887
C	-2.76915	-2.24903	-0.37367
C	-1.55914	-2.70754	0.04355
C	-0.50226	-1.80922	0.36993
O	0.53373	-2.44119	0.96928
O	-0.51879	2.49451	-0.89805
H	-1.62633	1.94773	1.03352
H	1.56999	-1.93436	-1.04024
H	3.86668	-2.72209	-1.01648
H	5.69541	-1.30096	-0.07672
H	5.16783	1.03052	0.60172
H	3.60817	2.90048	0.67650
H	1.38766	3.77901	-0.07075
H	-3.93188	2.70382	0.95246
H	-5.72043	1.24914	-0.01352
H	-5.14606	-1.08204	-0.65428
H	-3.56142	-2.93100	-0.66796
H	-1.34454	-3.76756	0.13781
H	1.20987	-1.80506	1.25570
H	-1.20276	1.87275	-1.19643

Molecule 2 (radical cation TS m062x)

C	-2.66953	1.42701	-0.05945
C	-2.02179	0.18043	0.06753
C	-0.61449	-0.05394	0.42164
C	0.58915	0.69917	0.09709
C	1.78881	-0.06508	-0.27090
C	1.70661	-1.34913	-0.85282
C	2.84786	-2.05850	-1.17969
C	4.12160	-1.51580	-0.95067
C	4.23212	-0.24141	-0.43310
C	3.07952	0.50426	-0.11137
C	3.19052	1.86437	0.30170
C	2.07694	2.64798	0.39780
C	0.78932	2.10785	0.18500
C	-3.97133	1.53239	-0.52607
C	-4.70029	0.39763	-0.88945
C	-4.13873	-0.84546	-0.66722
C	-2.83730	-0.97220	-0.15094
C	-2.39636	-2.26963	0.26700
C	-1.23790	-2.42219	0.96239
C	-0.37386	-1.31283	1.09173
O	0.67707	-1.52053	1.86783
O	-0.18701	3.02168	0.17256
H	-2.22986	2.32322	0.34921
H	0.73630	-1.77765	-1.08235
H	2.75154	-3.03917	-1.63440
H	5.01027	-2.08299	-1.20671
H	5.20518	0.21906	-0.28768
H	4.17810	2.28922	0.45675
H	2.13729	3.71138	0.60257
H	-4.42950	2.51463	-0.57885
H	-5.70898	0.48786	-1.27757
H	-4.70660	-1.75230	-0.85490
H	-3.05624	-3.11608	0.10029
H	-0.93724	-3.36268	1.40985
H	1.23155	-0.72041	1.95408
H	-0.86768	2.76716	-0.47472

Molecule 3 (neutral TS b3lyp)

C	2.54831	0.98878	0.69165
C	1.99120	-0.17094	0.08151
C	0.55558	-0.47390	0.05794
C	-0.53720	0.53044	0.00341
C	-1.95212	0.16783	-0.13695
C	-2.39600	-1.01096	-0.80145
C	-3.73445	-1.32757	-0.91924
C	-4.72854	-0.49156	-0.36227
C	-4.34887	0.70402	0.20405
C	-2.98046	1.08420	0.26809
C	-2.62039	2.41012	0.61870
C	-1.34541	2.84362	0.36076
C	-0.33796	1.93648	-0.06534
C	3.90632	1.24045	0.68672
C	4.80681	0.35088	0.05948
C	4.32287	-0.83634	-0.44158
C	2.93828	-1.15063	-0.37585
C	2.49414	-2.47222	-0.63527
C	1.23090	-2.83839	-0.24602
C	0.30227	-1.86544	0.21183
O	-0.84231	-2.24366	0.83497
O	0.82415	2.40169	-0.58633
H	1.90264	1.66032	1.23895
H	-1.66977	-1.64788	-1.29040
H	-4.02449	-2.22167	-1.46564
H	-5.77813	-0.76476	-0.43121
H	-5.09437	1.40865	0.56615
H	-3.37769	3.09858	0.98426
H	-1.09682	3.89127	0.48262
H	4.28579	2.12458	1.19323
H	5.87002	0.57394	0.03047

H	4.99984	-1.58624	-0.84477
H	3.18936	-3.20829	-1.03035
H	0.93109	-3.87861	-0.29293
C	1.12495	3.79600	-0.55783
H	0.39598	4.37372	-1.13791
H	1.16985	4.18339	0.46699
H	2.10892	3.88930	-1.02136
C	-1.19680	-3.62299	0.91585
H	-1.32937	-4.07072	-0.07621
H	-0.44995	-4.19443	1.47965
H	-2.14831	-3.64680	1.45020

Molecule 3 (radical cation TS b3lyp)

C	2.21503	-1.02508	-0.96325
C	1.89044	0.14135	-0.23906
C	0.49894	0.56662	0.02197
C	-0.56752	-0.38429	0.17054
C	-2.02717	-0.15478	0.08588
C	-2.71158	0.96388	0.60085
C	-4.08605	1.11006	0.44509
C	-4.84227	0.14960	-0.24195
C	-4.21781	-1.00775	-0.67456
C	-2.83457	-1.20256	-0.46563
C	-2.27541	-2.50167	-0.65510
C	-1.04398	-2.81312	-0.13185
C	-0.24103	-1.78399	0.40677
C	3.53422	-1.39441	-1.19069
C	4.59447	-0.61492	-0.69964
C	4.31083	0.57249	-0.04822
C	2.97365	0.98608	0.14701
C	2.68949	2.30433	0.60813
C	1.42475	2.82476	0.48334
C	0.35295	2.00950	0.04993
O	-0.80334	2.53081	-0.33741
O	0.82661	-2.02636	1.15776
H	1.42330	-1.63072	-1.39090
H	-2.17929	1.70555	1.17629
H	-4.57606	1.97727	0.87862
H	-5.90981	0.28721	-0.38478
H	-4.79017	-1.80879	-1.13444
H	-2.88997	-3.27848	-1.10126
H	-0.69816	-3.83901	-0.11187
H	3.74275	-2.28958	-1.76983
H	5.62322	-0.91973	-0.86649
H	5.11085	1.22845	0.28435
H	3.50520	2.93269	0.95451
H	1.24619	3.87190	0.69312
C	1.27929	-3.37709	1.37432
H	0.50177	-3.96666	1.86850
H	1.57199	-3.84445	0.42953
H	2.14711	-3.28176	2.02640
C	-1.03140	3.95331	-0.29103
H	-0.98813	4.32040	0.73850
H	-0.30345	4.47697	-0.91691
H	-2.03524	4.08916	-0.69321

Molecule 3 (neutral TS m062x)

C	2.60001	0.97535	0.62008
C	1.99701	-0.18526	0.05650
C	0.56257	-0.45791	0.09037
C	-0.52809	0.55463	0.02452
C	-1.92873	0.17864	-0.14372
C	-2.34051	-1.02197	-0.78956
C	-3.66536	-1.35204	-0.93826
C	-4.68271	-0.50740	-0.43706
C	-4.33078	0.70120	0.10452
C	-2.96765	1.08990	0.20340
C	-2.62982	2.42565	0.53878
C	-1.35406	2.86059	0.32172
C	-0.33017	1.94650	-0.05037
C	3.95648	1.19128	0.56288
C	4.81329	0.26068	-0.06644
C	4.28696	-0.92610	-0.50658
C	2.89837	-1.19934	-0.38616
C	2.41902	-2.52176	-0.57203
C	1.16740	-2.84146	-0.12867
C	0.27705	-1.82089	0.30462
O	-0.86162	-2.13842	0.96014
O	0.84060	2.40770	-0.53736
H	1.98745	1.67108	1.17626
H	-1.59018	-1.66722	-1.23329
H	-3.93139	-2.26402	-1.46535
H	-5.72634	-0.79121	-0.53118
H	-5.08939	1.40998	0.42747
H	-3.40626	3.11350	0.86164
H	-1.11052	3.91048	0.43468
H	4.37484	2.07655	1.03361
H	5.87883	0.45621	-0.13668
H	4.92919	-1.70799	-0.90430
H	3.08896	-3.28779	-0.95255
H	0.84079	-3.87458	-0.11861
C	1.19927	3.76772	-0.35610
H	0.55214	4.43002	-0.94052
H	1.16510	4.05618	0.70053
H	2.22345	3.85279	-0.72216
C	-1.34484	-3.47071	0.91869
H	-1.46113	-3.82566	-0.11188
H	-0.68453	-4.14940	1.46907
H	-2.32108	-3.44685	1.40412

Molecule 3 (radical cation TS m062x)

C	2.11918	-1.07179	-0.96146
C	1.85513	0.12892	-0.27025
C	0.48988	0.59493	0.03319
C	-0.58678	-0.33778	0.20931
C	-2.04409	-0.14494	0.06366
C	-2.78376	0.97656	0.48573
C	-4.14854	1.06780	0.26178
C	-4.84379	0.04911	-0.40080
C	-4.16769	-1.10723	-0.73298
C	-2.79306	-1.24063	-0.45691
C	-2.19923	-2.53830	-0.55049
C	-0.98634	-2.79388	0.02330
C	-0.22913	-1.70731	0.51934
C	3.41341	-1.48370	-1.22033
C	4.51078	-0.71229	-0.80547
C	4.28281	0.50254	-0.19520
C	2.96654	0.95115	0.04095
C	2.73735	2.28885	0.48234
C	1.48631	2.83345	0.42719
C	0.37400	2.02948	0.07383
O	-0.79591	2.56374	-0.20419
O	0.83436	-1.87240	1.27968
H	1.29517	-1.67396	-1.33248
H	-2.30336	1.76060	1.04918
H	-4.68268	1.94066	0.62420

H	-5.90659	0.14341	-0.59724
H	-4.69207	-1.95361	-1.16754
H	-2.78581	-3.34971	-0.97172
H	-0.61119	-3.80606	0.10746
H	3.57701	-2.40750	-1.76676
H	5.52314	-1.04983	-1.00116
H	5.10855	1.15239	0.08098
H	3.58727	2.90164	0.76877
H	1.33805	3.88388	0.64284
C	1.37884	-3.18178	1.47583
H	0.66488	-3.81281	2.01120
H	1.64394	-3.63275	0.51505
H	2.27374	-3.03280	2.07726
C	-1.01160	3.97378	-0.08073
H	-0.85744	4.29649	0.95203
H	-0.35085	4.51954	-0.75858
H	-2.05062	4.12800	-0.36842

Molecule 4 (neutral TS b3lyp)

C	-2.35523	-1.39593	0.69666
C	-2.00150	-0.15934	0.07870
C	-0.61660	0.31646	-0.04083
C	0.53837	-0.61328	-0.14689
C	1.96089	-0.22867	-0.11634
C	2.46224	0.99919	-0.62848
C	3.80482	1.32685	-0.57202
C	4.74192	0.44433	0.00361
C	4.31578	-0.80515	0.39917
C	2.95376	-1.18701	0.28194
C	2.58940	-2.55743	0.40545
C	1.35897	-2.95205	-0.03739
C	0.37286	-1.99621	-0.40537
C	-3.66679	-1.82227	0.78588
C	-4.71897	-1.04359	0.25839
C	-4.43172	0.20207	-0.25493
C	-3.09958	0.69114	-0.29593
C	-2.84178	2.05498	-0.58272
C	-1.60432	2.57152	-0.29688
C	-0.52800	1.72817	0.09107
O	0.59495	2.25762	0.63135
O	-0.70028	-2.57515	-1.01067
H	-1.58291	-2.00021	1.15559
H	1.79180	1.67484	-1.13791
H	4.14046	2.26829	-1.00009
H	5.79049	0.72123	0.07283
H	5.02710	-1.54827	0.75264
H	3.32725	-3.28302	0.73693
H	1.08610	-4.00024	-0.11820
H	-3.88631	-2.75982	1.29010
H	-5.74419	-1.39967	0.30832
H	-5.22988	0.86100	-0.58927
H	-3.64902	2.69937	-0.92014
H	-1.43725	3.63973	-0.36853
C	0.77243	3.67248	0.69796
H	0.78166	4.13058	-0.29803
H	-0.00326	4.14213	1.31331
H	1.74558	3.81857	1.17045
H	-1.35667	-1.90189	-1.25975

Molecule 4 (radical cation TS b3lyp)

C	2.79909	1.42946	0.21568
C	2.09338	0.21681	0.03443
C	0.66531	-0.02662	0.29469
C	-0.49475	0.83400	0.08160
C	-1.74925	0.21537	-0.38136
C	-1.76712	-0.96814	-1.15340
C	-2.95989	-1.54108	-1.57551
C	-4.19282	-0.95164	-1.25395
C	-4.20765	0.24185	-0.55249
C	-3.00300	0.85348	-0.13876
C	-3.01559	2.15326	0.44434
C	-1.84331	2.84320	0.60975
C	-0.59804	2.24564	0.31490
C	4.12627	1.57564	-0.17551
C	4.82611	0.51602	-0.76555
C	4.20946	-0.72123	-0.84306
C	2.88072	-0.90377	-0.40336
C	2.37051	-2.22995	-0.28198
C	1.18761	-2.47408	0.36552
C	0.36964	-1.38185	0.72894
O	-0.70696	-1.51753	1.49404
O	0.42974	3.11087	0.35448
H	2.37214	2.23123	0.80103
H	-0.83357	-1.42724	-1.45794
H	-2.93126	-2.44611	-2.17559
H	-5.12157	-1.40909	-1.58113
H	-5.14584	0.74489	-0.33452
H	-3.96748	2.62738	0.66656
H	-1.83172	3.87954	0.93135
H	4.62584	2.52192	0.01009
H	5.85250	0.64466	-1.09430
H	4.75436	-1.58823	-1.20625
H	2.98951	-3.05689	-0.61837
H	0.87688	-3.48727	0.58625
C	-1.15322	-2.82443	1.90796
H	-1.41802	-3.43361	1.03880
H	-0.38440	-3.32079	2.50678
H	-2.03746	-2.63935	2.51729
H	1.15720	2.80075	-0.21537

Molecule 4 (neutral TS m062x)

C	2.32970	-1.40293	-0.67839
C	1.99630	-0.15082	-0.08282
C	0.62258	0.33195	0.04360
C	-0.53149	-0.60171	0.15233
C	-1.95008	-0.23174	0.08906
C	-2.46791	0.99905	0.57633
C	-3.80688	1.31004	0.49522
C	-4.72583	0.40671	-0.07738
C	-4.28364	-0.84272	-0.43562
C	-2.92019	-1.20363	-0.29298
C	-2.54135	-2.57469	-0.37193
C	-1.32159	-2.94183	0.10398
C	-0.35222	-1.96030	0.45731
C	3.63195	-1.83097	-0.78581
C	4.69687	-1.04198	-0.29883
C	4.42469	0.20908	0.19321
C	3.09414	0.69788	0.25278
C	2.84606	2.06672	0.52451
C	1.60950	2.58128	0.25801
C	0.52836	1.72907	-0.09863
O	-0.59501	2.24702	-0.63367
O	0.71872	-2.49977	1.08759
H	1.54192	-2.01478	-1.10137
H	-1.80926	1.68332	1.09230
H	-4.15902	2.25289	0.90425

H	-5.77492	0.67141	-0.16634
H	-4.97991	-1.60264	-0.78165
H	-3.26799	-3.31446	-0.69558
H	-1.03264	-3.98208	0.21765
H	3.84063	-2.77892	-1.27272
H	5.71887	-1.40168	-0.36431
H	5.22808	0.87377	0.50086
H	3.66359	2.71109	0.83496
H	1.44115	3.64996	0.32021
C	-0.85989	3.63633	-0.52085
H	-0.82557	3.96944	0.52239
H	-0.15531	4.22360	-1.11875
H	-1.86801	3.77599	-0.91321
H	1.33425	-1.80356	1.37130

Molecule 4 (radical cation TS m062x)

C	2.81758	1.44052	0.08598
C	2.10325	0.22302	0.03887
C	0.68053	0.01532	0.32508
C	-0.47816	0.86179	0.07601
C	-1.70233	0.21590	-0.41185
C	-1.66853	-0.98006	-1.16125
C	-2.83482	-1.58857	-1.58919
C	-4.08773	-1.02362	-1.30142
C	-4.14681	0.17697	-0.62561
C	-2.96621	0.82106	-0.19869
C	-3.02201	2.12011	0.38595
C	-1.87409	2.82873	0.59783
C	-0.61020	2.25451	0.33894
C	4.13595	1.53219	-0.33001
C	4.82050	0.41364	-0.81587
C	4.19116	-0.81371	-0.77160
C	2.86632	-0.93454	-0.31065
C	2.34085	-2.24583	-0.09657
C	1.14902	-2.43777	0.53732
C	0.34608	-1.30767	0.81110
O	-0.76151	-1.38522	1.52108
O	0.41258	3.11212	0.45853
H	2.40923	2.30331	0.58787
H	-0.71403	-1.41716	-1.43758
H	-2.77484	-2.50456	-2.16870
H	-4.99680	-1.51187	-1.63643
H	-5.09967	0.66076	-0.42997
H	-3.99118	2.57034	0.58135
H	-1.88790	3.85863	0.93751
H	4.64463	2.48722	-0.24708
H	5.84472	0.49902	-1.16232
H	4.72016	-1.71771	-1.05915
H	2.95916	-3.09761	-0.36607
H	0.81894	-3.43019	0.81690
C	-1.34554	-2.66181	1.80313
H	-1.50362	-3.21571	0.87300
H	-0.71042	-3.22904	2.48784
H	-2.30100	-2.44164	2.27594
H	1.08516	2.91004	-0.21502

Molecule 5 (neutral TS b3lyp)

C	2.36231	1.28774	-0.62590
C	1.97768	0.02881	-0.08555
C	0.58383	-0.44826	-0.06698
C	-0.58382	0.44828	0.06697
C	-1.97768	-0.02881	0.08555
C	-2.36229	-1.28773	0.62593
C	-3.67880	-1.71632	0.62950
C	-4.70324	-0.91071	0.09320
C	-4.38794	0.36297	-0.33586
C	-3.05644	0.84479	-0.28554
C	-2.78521	2.23317	-0.44516
C	-1.57521	2.71253	-0.03397
C	-0.50321	1.84493	0.35675
C	3.67883	1.71632	-0.62946
C	4.70326	0.91069	-0.09317
C	4.38794	-0.36299	0.33586
C	3.05644	-0.84480	0.28552
C	2.78519	-2.23318	0.44514
C	1.57519	-2.71253	0.03394
C	0.50319	-1.84491	-0.35677
H	1.61516	1.91467	-1.09139
H	-1.61513	-1.91464	1.09143
H	-3.92254	-2.67860	1.07344
H	-5.73087	-1.26379	0.07525
H	-5.16884	1.04547	-0.66507
H	-3.57300	2.90576	-0.77432
H	-1.38778	3.78354	0.00748
H	3.92258	2.67861	-1.07337
H	5.73089	1.26376	-0.07521
H	5.16883	-1.04551	0.66506
H	3.57298	-2.90579	0.77428
H	1.38775	-3.78354	-0.00751
N	0.56169	2.47535	0.96810
H	0.34025	3.36075	1.40778
H	1.19832	1.90764	1.50963
N	-0.56170	-2.47533	-0.96812
H	-1.19838	-1.90761	-1.50960
H	-0.34028	-3.36072	-1.40781

Molecule 5 (radical cation TS b3lyp)

C	-2.69792	1.40148	0.07265
C	-2.03489	0.15409	0.07944
C	-0.61223	-0.08302	0.39997
C	0.58088	0.69448	0.08362
C	1.80757	-0.05179	-0.26703
C	1.76075	-1.31906	-0.89414
C	2.91804	-2.01893	-1.20365
C	4.18196	-1.48306	-0.90530
C	4.26404	-0.21984	-0.34390
C	3.09705	0.51757	-0.04954
C	3.17962	1.87560	0.39142
C	2.06308	2.65368	0.41318
C	0.76415	2.12865	0.10560
C	-4.02096	1.53531	-0.33516
C	-4.75748	0.42338	-0.75938
C	-4.17523	-0.83116	-0.66632
C	-2.85338	-0.99139	-0.20616
C	-2.37900	-2.31297	0.09274
C	-1.22665	-2.49094	0.78820
C	-0.37920	-1.36716	1.05387
H	-2.21266	2.26994	0.48984
H	0.80203	-1.74682	-1.16592
H	2.84048	-2.98368	-1.69730
H	5.08407	-2.03948	-1.14214
H	5.23050	0.23772	-0.14824
H	4.15277	2.30411	0.61493
H	2.13188	3.71789	0.62345
H	-4.48584	2.51658	-0.30022
H	-5.78162	0.53351	-1.10274

H	-4.74789	-1.72254	-0.90853
H	-3.01407	-3.16036	-0.15086
H	-0.92400	-3.46710	1.15596
N	-0.20938	3.04970	-0.07816
H	0.07503	4.02343	-0.09347
H	-0.98671	2.83705	-0.68724
N	0.61918	-1.53209	1.92794
H	1.26180	-0.78399	2.15494
H	0.82436	-2.44915	2.30835

Molecule 5 (neutral TS m062x)

C	-2.34148	-1.28276	-0.62099
C	-1.96806	-0.02566	-0.07165
C	-0.58179	0.45347	-0.05763
C	0.58181	-0.45323	0.05783
C	1.96814	0.02570	0.07158
C	2.34186	1.28278	0.62072
C	3.65096	1.71399	0.63032
C	4.67986	0.91340	0.09597
C	4.37306	-0.35686	-0.33226
C	3.04350	-0.83888	-0.28864
C	2.77696	-2.22963	-0.44008
C	1.57289	-2.70549	-0.02788
C	0.49742	-1.83475	0.35280
C	-3.65051	-1.71416	-0.63089
C	-4.67963	-0.91380	-0.09663
C	-4.37311	0.35645	0.33183
C	-3.04361	0.83868	0.28851
C	-2.77730	2.22945	0.44024
C	-1.57322	2.70555	0.02835
C	-0.49756	1.83505	-0.35233
H	-1.58551	-1.90138	-1.08522
H	1.58609	1.90156	1.08505
H	3.89058	2.67339	1.07996
H	5.70493	1.27067	0.08382
H	5.15533	-1.03834	-0.65841
H	3.56747	-2.90047	-0.76471
H	1.38046	-3.77484	0.01538
H	-3.88989	-2.67355	-1.08068
H	-5.70464	-1.27123	-0.08471
H	-5.15554	1.03776	0.65794
H	-3.56795	2.90012	0.76486
H	-1.38094	3.77494	-0.01467
N	-0.56762	-2.46890	0.95895
H	-0.31853	-3.32629	1.43830
H	-1.20218	-1.89008	1.49350
N	0.56744	2.46949	-0.95825
H	1.20210	1.89088	-1.49290
H	0.31826	3.32694	-1.43743

Molecule 5 (radical cation TS m062x)

C	-2.69138	1.41949	-0.04326
C	-2.02438	0.18257	0.07483
C	-0.61405	-0.02164	0.43851
C	0.58722	0.72693	0.09821
C	1.76783	-0.06466	-0.28727
C	1.64324	-1.33994	-0.88336
C	2.75605	-2.08427	-1.22494
C	4.04941	-1.58757	-0.99509
C	4.20115	-0.32324	-0.46455
C	3.07538	0.45542	-0.12948
C	3.22583	1.80976	0.31009
C	2.14423	2.62100	0.40131
C	0.81315	2.14073	0.15495
C	-3.99509	1.50948	-0.50502
C	-4.70605	0.36418	-0.87414
C	-4.12031	-0.87122	-0.67002
C	-2.81665	-0.98206	-0.15929
C	-2.34099	-2.28355	0.23179
C	-1.20313	-2.41594	0.94618
C	-0.36910	-1.26518	1.15012
H	-2.23793	2.31599	0.34668
H	0.65609	-1.73355	-1.10457
H	2.62446	-3.05540	-1.69211
H	4.91855	-2.18033	-1.26119
H	5.18981	0.10296	-0.31679
H	4.22407	2.20015	0.48708
H	2.25702	3.67717	0.62968
H	-4.46948	2.48453	-0.55464
H	-5.71764	0.43958	-1.25873
H	-4.67406	-1.78487	-0.86809
H	-2.96956	-3.14528	0.02548
H	-0.88724	-3.36616	1.36589
N	-0.14936	3.08407	0.07873
H	0.16091	4.05008	0.10328
H	-0.92478	2.92553	-0.54907
N	0.64199	-1.37271	2.00334
H	1.25589	-0.59217	2.20609
H	0.85143	-2.25945	2.44902

Molecule 6 (neutral TS b3lyp)

C	-0.50036	3.21874	0.68003
C	-0.50036	3.21874	-0.68003
C	-0.26270	2.01026	-1.38403
C	-0.33572	0.72952	-0.73921
C	-0.33572	0.72952	0.73921
C	-0.26270	2.01026	1.38403
C	0.20438	2.16663	-2.73162
C	-0.20508	-0.41293	-1.67745
C	0.47074	-0.21009	-2.93357
C	0.68960	1.11143	-3.43052
C	0.79351	-1.30701	-3.76821
H	1.35197	-1.10611	-4.67940
C	0.35548	-2.58454	-3.48241
C	-0.49475	-2.76010	-2.38063
C	-0.76755	-1.70368	-1.52477
H	0.26005	3.17578	-3.13110
H	-0.56540	4.14652	1.24219
H	-0.56540	4.14652	-1.24219
H	1.16105	1.23869	-4.40151
H	0.59999	-3.41688	-4.13660
H	-0.97052	-3.72084	-2.20246
H	-1.52249	-1.86650	0.78155
C	0.20438	2.16663	2.73162
H	0.26005	3.17578	3.13110
C	0.68960	1.11143	3.43052
H	1.16105	1.23869	4.40151
C	-0.20508	-0.41293	1.67745
C	0.47074	-0.21009	2.93357
C	0.79351	-1.30701	3.76821
H	1.35197	-1.10611	4.67940
C	0.35548	-2.58454	3.48241
H	0.59999	-3.41688	4.13660
C	-0.49475	-2.76010	2.38063
H	-0.97052	-3.72084	2.20246
C	-0.76755	-1.70368	1.52477

H	-1.52249	-1.86650	-0.78155

Molecule 6 (radical cation TS b3lyp)

C	-0.67708	3.24911	-0.54872
C	0.67708	3.24911	-0.54872
C	1.37577	2.03285	-0.27608
C	0.72074	0.73871	-0.37034
C	-0.72074	0.73871	-0.37034
C	-1.37577	2.03285	-0.27607
C	2.68017	2.16590	0.24468
C	1.64092	-0.41524	-0.22929
C	2.85834	-0.23017	0.51593
C	3.34769	1.07837	0.76083
C	3.68214	-1.33218	0.84890
H	4.56524	-1.14802	1.45419
C	3.41138	-2.60168	0.36279
C	2.35373	-2.74918	-0.53563
C	1.50765	-1.67456	-0.83551
H	3.10529	3.16165	0.32485
H	-1.24733	4.16951	-0.62785
H	1.24733	4.16951	-0.62785
H	4.29471	1.20210	1.27842
H	4.04939	-3.44213	0.61685
H	2.18611	-3.69973	-1.03349
H	-0.80954	-1.81491	-1.63842
C	-2.68017	2.16590	0.24468
H	-3.10529	3.16165	0.32486
C	-3.34769	1.07837	0.76084
H	-4.29471	1.20210	1.27843
C	-1.64092	-0.41524	-0.22929
C	-2.85834	-0.23017	0.51593
C	-3.68214	-1.33218	0.84891
H	-4.56524	-1.14803	1.45419
C	-3.41138	-2.60168	0.36279
H	-4.04939	-3.44213	0.61685
C	-2.35373	-2.74918	-0.53563
H	-2.18611	-3.69973	-1.03349
C	-1.50765	-1.67456	-0.83551
H	0.80954	-1.81491	-1.63842

Molecule 6 (neutral TS m062x)

C	-3.21712	-0.49871	0.67842
C	-3.21712	-0.49871	-0.67842
C	-2.00567	-0.27307	-1.37842
C	-0.73776	-0.35359	-0.73567
C	-0.73776	-0.35359	0.73567
C	-2.00567	-0.27307	1.37842
C	-2.15812	0.20827	-2.72437
C	0.40897	-0.21224	-1.66197
C	0.21612	0.48093	-2.89703
C	-1.10411	0.70853	-3.40239
C	1.31428	0.81191	-3.72130
H	1.11840	1.38641	-4.62296
C	2.58493	0.36639	-3.43525
C	2.74967	-0.50636	-2.35147
C	1.69028	-0.78998	-1.51049
H	-3.16584	0.26652	-3.12585
H	-4.14376	-0.55415	1.24267
H	-4.14376	-0.55415	-1.24267
H	-1.22082	1.19523	-4.36667
H	3.42157	0.61774	-4.07961
H	3.70473	-0.99407	-2.18139
H	1.83694	-1.57019	0.79061
C	-2.15812	0.20827	2.72437
H	-3.16584	0.26652	3.12585
C	-1.10411	0.70853	3.40239
H	-1.22082	1.19523	4.36667
C	0.40897	-0.21224	1.66197
C	0.21612	0.48093	2.89703
C	1.31428	0.81191	3.72130
H	1.11840	1.38641	4.62296
C	2.58493	0.36639	3.43525
H	3.42157	0.61774	4.07961
C	2.74967	-0.50636	2.35147
H	3.70473	-0.99407	2.18139
C	1.69028	-0.78998	1.51049
H	1.83694	-1.57019	-0.79061

Molecule 6 (radical cation TS m062x)

C	0.56409	3.24899	-0.67445
C	0.56409	3.24899	0.67445
C	0.28607	2.02935	1.36910
C	0.39108	0.74191	0.71474
C	0.39108	0.74191	-0.71474
C	0.28607	2.02935	-1.36910
C	-0.25945	2.16005	2.65958
C	0.24033	-0.41570	1.62444
C	-0.53162	-0.23803	2.81512
C	-0.79175	1.06962	3.30402
C	-0.87078	-1.33624	3.63247
H	-1.49330	-1.15522	4.50391
C	-0.37008	-2.59817	3.36928
C	0.55114	-2.73705	2.33267
C	0.86101	-1.66374	1.49628
H	-0.35029	3.15419	3.08559
H	0.64354	4.16633	-1.24909
H	0.64354	4.16633	1.24909
H	-1.33500	1.18478	4.23766
H	-0.62763	-3.44102	4.00135
H	1.06080	-3.68282	2.17838
H	1.68166	-1.79212	-0.81660
C	-0.25945	2.16005	-2.65958
H	-0.35029	3.15419	-3.08559
C	-0.79175	1.06962	-3.30402
H	-1.33500	1.18478	-4.23766
C	0.24033	-0.41570	-1.62444
C	-0.53162	-0.23803	-2.81512
C	-0.87078	-1.33624	-3.63247
H	-1.49330	-1.15522	-4.50391
C	-0.37008	-2.59817	-3.36928
H	-0.62763	-3.44102	-4.00135
C	0.55114	-2.73705	-2.33267
H	1.06080	-3.68282	-2.17838
C	0.86101	-1.66374	-1.49628
H	1.68166	-1.79212	0.81660

Molecule 7 (neutral TS b3lyp)

C	1.19880	3.53611	-0.77362
C	2.31137	3.03489	-0.17219
C	2.34493	1.66926	0.22254
C	1.32481	0.76357	-0.19602
C	0.00004	1.34675	-0.50932
C	0.00007	2.76213	-0.77432
C	3.41133	1.23395	1.07992
C	1.75875	-0.63835	-0.17202
C	2.76476	-1.05439	0.76394

C	3.52759	-0.06588	1.46274
C	3.14105	-2.41727	0.83799
H	3.86173	-2.71296	1.59676
C	2.68666	-3.33240	-0.09261
C	1.88411	-2.88043	-1.15520
C	1.43611	-1.56994	-1.18391
H	4.09019	1.99304	1.46060
H	1.14642	4.57764	-1.07863
H	3.17567	3.66118	0.03340
H	4.28211	-0.38319	2.17780
H	3.00708	-4.36983	-0.04725
H	1.62590	-3.55355	-1.96832
C	-1.19861	3.53617	-0.77361
H	-1.14619	4.57770	-1.07862
C	-2.31120	3.03501	-0.17216
H	-3.17547	3.66134	0.03344
C	-1.32476	0.76363	-0.19602
C	-2.34483	1.66938	0.22255
C	-3.41124	1.23412	1.07995
H	-4.09006	1.99325	1.46064
C	-3.52757	-0.06570	1.46276
H	-4.28210	-0.38298	2.17783
C	-2.76480	-1.05425	0.76394
C	-1.75878	-0.63827	-0.17203
H	0.90216	-1.21844	-2.05295
C	-3.14118	-2.41711	0.83799
H	-3.86187	-2.71276	1.59676
C	-1.43621	-1.56987	-1.18393
H	-0.90225	-1.21840	-2.05297
C	-2.68686	-3.33226	-0.09263
H	-3.00734	-4.36967	-0.04728
C	-1.88429	-2.88034	-1.15522
H	-1.62613	-3.55346	-1.96835

Molecule 7 (radical cation TS b3lyp)

C	1.19965	3.53341	-0.78576
C	2.31397	3.02765	-0.16677
C	2.33567	1.67867	0.24580
C	1.30503	0.76868	-0.20899
C	0.00000	1.35932	-0.53085
C	-0.00000	2.76990	-0.79398
C	3.36674	1.22326	1.12505
C	1.71284	-0.62569	-0.19248
C	2.68876	-1.05985	0.76597
C	3.45041	-0.08682	1.49110
C	3.03079	-2.42205	0.84348
H	3.72541	-2.74114	1.61553
C	2.56326	-3.33612	-0.10007
C	1.79365	-2.87742	-1.16933
C	1.37884	-1.54438	-1.21268
H	4.05250	1.96379	1.52634
H	1.16362	4.57085	-1.10434
H	3.17234	3.65802	0.04611
H	4.18168	-0.42491	2.21940
H	2.86234	-4.37834	-0.04258
H	1.53300	-3.54452	-1.98500
C	-1.19965	3.53341	-0.78575
H	-1.16362	4.57085	-1.10434
C	-2.31397	3.02765	-0.16677
H	-3.17235	3.65802	0.04611
C	-1.30503	0.76868	-0.20899
C	-2.33567	1.67867	0.24580
C	-3.36674	1.22326	1.12505
H	-4.05251	1.96379	1.52634
C	-3.45041	-0.08682	1.49111
H	-4.18168	-0.42491	2.21940
C	-2.68876	-1.05986	0.76597
C	-1.71284	-0.62569	-0.19248
H	0.91957	-1.16745	-2.11288
C	-3.03079	-2.42205	0.84348
H	-3.72540	-2.74114	1.61553
C	-1.37884	-1.54438	-1.21268
H	-0.91957	-1.16745	-2.11288
C	-2.56326	-3.33612	-0.10007
H	-2.86234	-4.37834	-0.04258
C	-1.79365	-2.87742	-1.16933
H	-1.53300	-3.54452	-1.98500

Molecule 7 (neutral TS m062x)

C	1.19715	3.54188	-0.75204
C	2.30450	3.03268	-0.15838
C	2.33314	1.66091	0.21528
C	1.31690	0.77222	-0.20659
C	-0.00014	1.35881	-0.52231
C	-0.00027	2.76586	-0.76669
C	3.39741	1.21432	1.07365
C	1.72964	-0.63320	-0.17599
C	2.71422	-1.05906	0.76118
C	3.49205	-0.07951	1.46114
C	3.07004	-2.42393	0.83957
H	3.78066	-2.72851	1.60352
C	2.60392	-3.33049	-0.08777
C	1.82162	-2.86709	-1.15867
C	1.40520	-1.55160	-1.19504
H	4.08027	1.96840	1.45590
H	1.14285	4.58697	-1.04264
H	3.17121	3.65164	0.05686
H	4.23738	-0.40895	2.17962
H	2.90422	-4.37265	-0.03759
H	1.56163	-3.53575	-1.97392
C	-1.19783	3.54165	-0.75205
H	-1.14372	4.58676	-1.04265
C	-2.30510	3.03226	-0.15841
H	-3.17193	3.65106	0.05680
C	-1.31707	0.77199	-0.20658
C	-2.33349	1.66049	0.21526
C	-3.39770	1.21369	1.07360
H	-4.08071	1.96763	1.45582
C	-3.49210	-0.08015	1.46109
H	-4.23739	-0.40974	2.17954
C	-2.71405	-1.05956	0.76117
C	-1.72952	-0.63351	-0.17597
H	0.89799	-1.18092	-2.07251
C	-3.06959	-2.42450	0.83955
H	-3.78018	-2.72923	1.60346
C	-1.40483	-1.55184	-1.19499
H	-0.89766	-1.18104	-2.07243
C	-2.60323	-3.33097	-0.08776
H	-2.90330	-4.37320	-0.03758
C	-1.82097	-2.86742	-1.15863
H	-1.56079	-3.53603	-1.97386

Molecule 7 (radical cation TS m062x)

C	1.19517	3.53066	-0.77924
C	2.30425	3.01922	-0.15672
C	2.31856	1.67133	0.24357
C	1.29441	0.77092	-0.22184

C	-0.00012	1.36189	-0.55178
C	-0.00025	2.76603	-0.80305
C	3.34139	1.20901	1.13553
C	1.68856	-0.62313	-0.20032
C	2.64048	-1.06265	0.76742
C	3.40466	-0.09345	1.50413
C	2.97008	-2.42113	0.84839
H	3.65521	-2.74618	1.62605
C	2.50073	-3.32764	-0.09819
C	1.75242	-2.86314	-1.17707
C	1.35894	-1.52889	-1.22763
H	4.02238	1.94864	1.54542
H	1.15988	4.57080	-1.08855
H	3.16107	3.64623	0.07020
H	4.12005	-0.44178	2.24304
H	2.78646	-4.37268	-0.03694
H	1.49346	-3.52854	-1.99415
C	-1.19593	3.53044	-0.77922
H	-1.16091	4.57044	-1.08898
C	-2.30474	3.01899	-0.15634
H	-3.16167	3.64582	0.07066
C	-1.29445	0.77086	-0.22158
C	-2.31869	1.67109	0.24407
C	-3.34106	1.20852	1.13634
H	-4.02216	1.94793	1.54645
C	-3.40410	-0.09407	1.50471
H	-4.11945	-0.44260	2.24356
C	-2.64000	-1.06308	0.76767
C	-1.68842	-0.62322	-0.20023
H	0.91430	-1.13968	-2.13069
C	-2.96967	-2.42155	0.84815
H	-3.65470	-2.74690	1.62577
C	-1.35915	-1.52845	-1.22804
H	-0.91514	-1.13875	-2.13123
C	-2.50048	-3.32772	-0.09890
H	-2.78619	-4.37279	-0.03796
C	-1.75244	-2.86284	-1.17776
H	-1.49341	-3.52800	-1.99500

5. References

[1] Gaussian 09, Revision D.01, M. J. Frisch, G. W. Trucks, H. B. Schlegel, G. E. Scuseria, M. A. Robb, J. R. Cheeseman, G. Scalmani, V. Barone, B. Mennucci, G. A. Petersson, H. Nakatsuji, M. Caricato, X. Li, H. P. Hratchian, A. F. Izmaylov, J. Bloino, G. Zheng, J. L. Sonnenberg, M. Hada, M. Ehara, K. Toyota, R. Fukuda, J. Hasegawa, M. Ishida, T. Nakajima, Y. Honda, O. Kitao, H. Nakai, T. Vreven, J. A. Montgomery, Jr., J. E. Peralta, F. Ogliaro, M. Bearpark, J. J. Heyd, E. Brothers, K. N. Kudin, V. N. Staroverov, R. Kobayashi, J. Normand, K. Raghavachari, A. Rendell, J. C. Burant, S. S. Iyengar, J. Tomasi, M. Cossi, N. Rega, J. M. Millam, M. Klene, J. E. Knox, J. B. Cross, V. Bakken, C. Adamo, J. Jaramillo, R. Gomperts, R. E. Stratmann, O. Yazyev, A. J. Austin, R. Cammi, C. Pomelli, J. W. Ochterski, R. L. Martin, K. Morokuma, V. G. Zakrzewski, G. A. Voth, P. Salvador, J. J. Dannenberg, S. Dapprich, A. D. Daniels, Ö. Farkas, J. B. Foresman, J. V. Ortiz, J. Cioslowski, and D. J. Fox, Gaussian, Inc., Wallingford CT, **2009**.

[2] (a) A. D. Becke, J. Chem. Phys. **1993**, *98*, 5648–5652; (b) C. Lee, W. Yang, R. G. Parr, Phys. Rev. B **1988**, *37*, 785–789; (c) S. H. Vosko, L. Wilk, M. Nusair, Can. J. Phys. **1980**, *58*, 1200–1211; (d) P. J. Stephens, F. J. Devlin, C. F. Chabalowski, M. J. Frisch, J. Phys. Chem. **1994**, *98*, 11623–11627.

[3] Y. Zhao, D. Truhlar, *Theor. Chem. Acc.,* **2008**, *120*, 215.

[4] Grimme, S., Ehrlich, S., Goerigk L. *J. Comp. Chem.,* **2011**, *32,* 1456–1465.

[5] (a) A. Schaefer, H. Horn and R. Ahlrichs, *J. Chem. Phys.*, **1992**, *97*, 2571; (b) A. Schaefer, C. Huber, and R. Ahlrichs, *J. Chem. Phys*., **1994**, *100*, 5829; (c) F. Weigend and R. Ahlrichs, *Phys. Chem. Chem. Phys.*, **2005**, 7, 3297.

[6] Mennucci, B., Cancès, E. & Tomasi, J. *J. Phys. Chem. B*, ***1997***, *101*, 10506–10517.

[7] K. Fukui, *Acc. Chem. Res.*, **1981**, *14*, 363–368

[8] A. T. B. Gilbert, IQmol molecular viewer, Available at: http://iqmol.org.

[9] S. Grimme, *Chem. Eur. J.,* **2012**, *18*, 9955.

[10] I. Funes-Ardoiz and R. S. Paton, (2016) GoodVibes v2.0.2. http://doi.org/10.5281/zenodo.595246
